# Multi-chamber three-dimensional myocardial strain assessment by computed tomography: a comparison with speckle tracking echocardiography and association with pulmonary hypertension in severe aortic stenosis

**DOI:** 10.3389/fcvm.2026.1774091

**Published:** 2026-05-13

**Authors:** Vitaliy Androshchuk, Edouard Long, Charles Sillett, Omar Chehab, Natalie Montarello, Joshua Wilcox, Marina Strocchi, Benedict McDonaugh, Jonathon Leipsic, Ronak Rajani, Bernard Prendergast, Steven Niederer, Tiffany Patterson, Simon Redwood

**Affiliations:** 1 School of Cardiovascular Medicine & Sciences, Faculty of Life Sciences & Medicine, King’s College London, London, United Kingdom; 2 School of Biomedical Engineering and Imaging Sciences, Faculty of Life Sciences & Medicine, King’s College London, London, United Kingdom; 3 National Heart and Lung Institute, Imperial College London, London, United Kingdom; 4 Cardiovascular Directorate, St Thomas’ Hospital, London, United Kingdom; 5 Centre for Cardiovascular Innovation and Center for Heart Valve Innovation, Division of Cardiology and Department of Radiology, St. Paul’s and Vancouver General Hospital, University of British Columbia, Vancouver, BC, Canada; 6 Cleveland Clinic London, London, United Kingdom

**Keywords:** aortic stenosis, cardiac computed tomography angiography, myocardial strain, pulmonary hypertension, speckle-tracking transthoracic echocardiography

## Abstract

**Background:**

Myocardial strain imaging is a robust tool for evaluating extra-valvular remodelling in aortic stenosis (AS). Multi-phase cardiac computed tomography (CT) angiography acquired for transcatheter aortic valve implantation (TAVI) planning enables a novel three-dimensional (3D), geometry-independent strain assessment beyond two-dimensional (2D) transthoracic echocardiography (TTE). This study evaluates the agreement and reproducibility of CT- and TTE-derived longitudinal strain and examines its association with pulmonary hypertension (PH) in significant AS.

**Methods:**

Left ventricular global longitudinal strain (LV-GLS), left atrial global longitudinal reservoir strain (LA-LS), right ventricular global longitudinal strain (RV-GLS), and RV free-wall longitudinal strain (RV-FWLS) were determined using 2D TTE and CT-based 3D motion tracking in patients with severe AS undergoing TAVI evaluation. Patients with PH were defined using guideline-directed TTE criteria for high probability PH (H-PH: *n* = 43, 46.2%) and compared with those at low probability (L-PH: *n* = 50, 53.8%).

**Results:**

Agreement between CT and TTE was strong for LV-GLS (r = 0.837), RV-GLS (r = 0.853), and RV-FWLS (r = 0.780) and moderate for LA-LS (r = 0.677) (all *p* < 0.001). Peak longitudinal strain on both TTE and CT was significantly reduced in H-PH compared with L-PH (*p* < 0.001). Optimal strain cutoff values for identifying H-PH were lower on CT than on TTE (LV-GLS: −16.6% vs. −17.9%; LA-LS: 10.2% vs. 14.4%; RV-GLS: −15.3% vs. −20.1%; RV-FWLS: −18.0% vs. −21.1%). In an inter-modality comparison, it was found that TTE-derived LV-GLS was superior to CT-derived LV-GLS for detecting H-PH [AUC: 0.94 [95% CI 0.89–0.99] vs. 0.85 [95% CI 0.78–0.93], *p* = 0.013], whereas differences for LA-LS, RV-GLS, and RV-FWLS were non-significant (all *p* > 0.05). TTE- and CT-derived strain measurements showed excellent reproducibility (ICC > 0.9).

**Conclusion:**

TAVI CT is a promising tool for 3D longitudinal strain assessment and a valuable adjunct to TTE for quantifying extra-valvular remodelling associated with AS progression to PH. Further studies are warranted to evaluate the prognostic value of multi-chamber CT-derived 3D strain in AS.

## 1 Introduction

Aortic stenosis (AS) is the most common valvular heart disease (VHD) in developed countries, which is now predominantly treated with transcatheter aortic valve implantation (TAVI) (1). Improved detection of structural and functional cardiopulmonary abnormalities associated with AS progression can enhance risk stratification and determine the optimal timing of intervention (2). Myocardial strain imaging integrates both volumetric and geometric cardiac properties and is increasingly recognised as a tool for a more precise assessment of extra-valvular remodelling. Global longitudinal strain has emerged as a sensitive biomarker for detecting early deformation abnormalities in AS, preceding overt reduction in ejection fraction (EF) (3). Moreover, impaired longitudinal strain of the left ventricle (LV), left atrium (LA), and right ventricle (RV) are associated with increased mortality in patients with AS undergoing TAVI (4).

Owing to its widespread availability and lack of ionising radiation, two-dimensional (2D) speckle-tracking transthoracic echocardiography (TTE) is the most common technique for performing a routine strain analysis. However, TTE variability can be substantial due to operator experience, vendor-specific settings, suboptimal image quality, apical foreshortening, and angle of incidence effect. More recently, strain analysis has become feasible with the use of 2D reconstructed planes from four-dimensional (4D) retrospective electrocardiography (ECG)-gated cardiac computed tomography angiography (CT) (5). To overcome the inherent limitations of 2D strain analysis in anatomically complex myocardial chambers, whole-chamber CT-derived three-dimensional (3D) strain can provide a truly geometry-independent characterisation of tissue biomechanics (6–8). Strain measurement on CT benefits from high isotopic spatial resolution and a wide field of view, allowing improved endocardial border visualisation and reproducible measurements. Given that CT is the gold standard for TAVI planning, extraction of additional strain parameters could enhance its value for risk stratification and outcome prediction without additional imaging, contrast administration, or radiation exposure.

Data comparing multi-chamber CT-derived 3D strain with conventional 2D TTE remain scarce. Furthermore, there is limited evidence on strain alterations in AS patients with pulmonary hypertension—a clinically important subgroup at increased risk of adverse outcomes following TAVI (9). Against this background, the primary objective of the present study is to determine the correlation, agreement, and reproducibility of 3D CT- vs. 2D TTE-derived strain for the LV, LA, and RV in patients with severe AS awaiting TAVI. The secondary objective is to compare the associations of 3D CT- and 2D TTE-derived strain with PH in AS.

## 2 Materials and methods

### 2.1 Study design and population

This was a prospective cohort study of adult patients (>18 years) with severe symptomatic AS undergoing TAVI evaluation. All participants underwent clinically indicated TTE and CT on the same day. Severe AS was defined as an aortic valve area (AVA) < 1 cm^2^ (or an indexed AVA ≤0.6 cm^2^/m^2^) and/or a mean aortic valve gradient ≥40 mmHg and/or a peak aortic jet velocity ≥4 m/s, according to current guidelines (10). PH was defined according to TTE criteria, using peak tricuspid regurgitation jet velocity (TR Vmax) and additional parameters suggestive of PH, in line with guideline recommendations (11). Patients were divided into two groups for comparison: high probability of PH (H-PH) and low probability of PH (L-PH). The exclusion criteria were as follows: at least moderate VHD other than AS, previous valve surgery, implanted permanent pacemaker, significant lung disease, left ventricular EF (LV-EF) < 50%, significant coronary artery disease, prior myocardial infarction, insufficient TR to estimate pulmonary artery systolic pressure, and poor image quality. Baseline clinical characteristics, serum biomarkers, and standard TTE parameters were obtained at the time of recruitment. The study protocol complied with the declaration of Helsinki and was approved by the National Ethics Review Board (Integrated Research Application System Reference: 319698). The final study population consisted of 93 patients recruited between June 2023 and November 2024. All patients provided written informed consent.

### 2.2 TAVI CT acquisition

CT image acquisition was performed over the entire cardiac cycle using a dual-source scanner (SOMATOM Force, Siemens Healthcare) and a dedicated research protocol (12). Scan parameters were as follows: reference tube voltage 100–120 kV and reference tube-current-time product 125–300 mAs, according to body mass index (BMI), gantry rotation time 250 ms, slice collimation 128 × 0.6 mm, and pitch value 0.2–0.45, depending on heart rate. A triphasic contrast bolus consisted of 50 mL of contrast (5 mL/s), 100 mL of contrast-saline mix (1:1) (5 mL/s), and 50 mL of saline flush (3 mL/s). Data acquisition was initiated when the threshold of 110 Hounsfield units was reached in the proximal descending aorta, followed by a 5 s delay. Spiral acquisition was performed during an inspiratory breath hold in a cranio-caudal direction with no tube current modulation. Images were reconstructed with a slice thickness of 1 mm in 5% increments to obtain a total of 20 reconstructions per cardiac cycle (0%–100% of RR interval) for feature tracking.

### 2.3 Measurement of 3D CT-derived myocardial strain and volume

**Figure 1:**
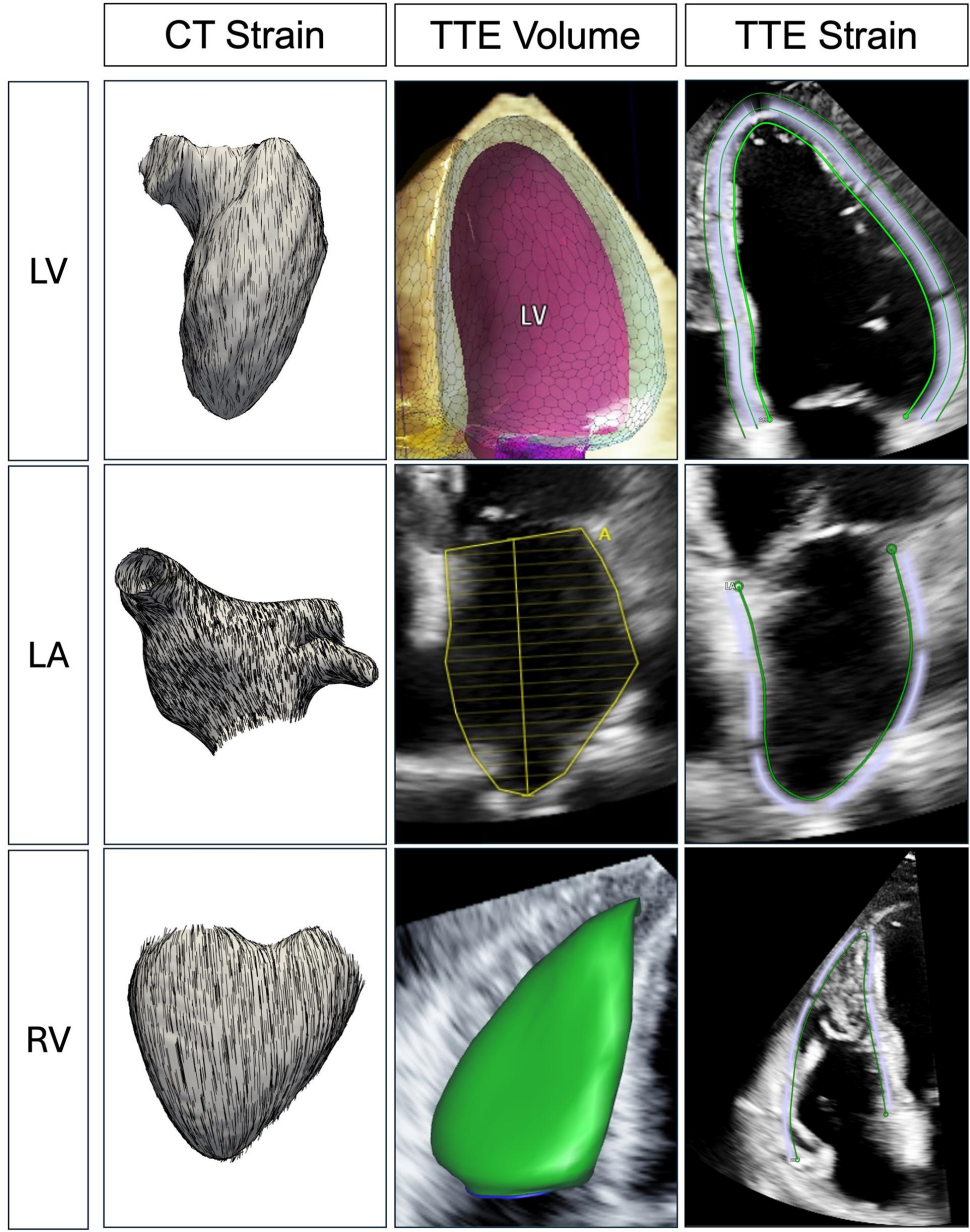
Illustration of multi-chamber CT-derived strain and TTE-derived volume and strain assessment. Longitudinal strain was measured using ventricular end-diastole as the reference point on both CT and TTE. For TTE, LV strain was averaged from apical four-, three-, and two-chamber views, whereas RV strain was assessed from the RV-focused apical four-chamber view. A full-volume dataset for the LV and RV was acquired from apical four-chamber and RV-focused views to quantify EF, whereas LA volume was measured at ventricular end-systole using the biplane method. CT, computed tomography; EF, ejection fraction; LA, left atrium; LV, left ventricle; RV, right ventricle; TTE, transthoracic echocardiography.

Multi-phase TAVI CT was used to evaluate 3D myocardial strain according to previously validated methodology (Supplementary Methodology in the Supplementary Material) (6, 7). Patient-specific models of myocardial chambers were semi-automatically constructed from CT images at end-diastole using CemrgApp, with manual corrections to endocardial borders if necessary (13, 14). Anatomical landmarks were selected on end-diastolic images of the LV, LA, and RV to define longitudinal axes. Cardiac model deformation was automatically tracked throughout the cardiac cycle using 3D feature tracking (Figure 1). Longitudinal strain was defined as the percentage change in length at peak systole along the longitudinal axes and tangential to the endocardial surface, with ventricular end-diastole used as the zero reference. CT-derived peak longitudinal strain was measured for: (1) global LV (LV-GLSCT); (2) global LA (LA-LSCT); (3) global RV (RV-GLSCT); and (4) RV free-wall (RV-FWLSCT). 3D end-diastolic volume (EDV), end-systolic volume (ESV), and EF were calculated from myocardial chamber segmentations at end-diastole (0%) and peak systole.

### 2.4 TTE acquisition and analysis

All TTE images were acquired using an EPIQ CVx v7.0 system (Philips Healthcare, Netherlands) and X5–1c transducer. A 2D strain analysis was performed semi-automatically using TomTec Arena (2D Cardiac Performance Analysis, Tomtec Imaging Systems GmbH, Unterschleißheim, Germany) according to current recommendations, with the reference point placed at end-diastole (15). LV strain was measured from apical four-, three-, and two-chamber views; RV strain from the RV-focused apical four-chamber view; and LA strain from apical four- and two-chamber views (Figure 1). Care was taken to avoid apical foreshortening and achieve the highest frame rate possible (minimum ≥60 frames/s). Endocardial borders were reviewed at end-diastole and end-systole and manually corrected if necessary. In sinus rhythm, strain was measured during a single cardiac cycle selected from three-beat cine loops with similar RR intervals. In atrial fibrillation (AF), strain was averaged over three separate cardiac cycles. TTE-derived peak longitudinal strain was measured in absolute values, with ventricular end-diastole as the zero reference point, for: (1) global LV (LV-GLSTTE); (2) global LA (LA-LSTTE); (3) global RV (RV-GLSTTE); and (4) RV free-wall (RV-FWLSTTE).

Full-volume 3D datasets of the LV and RV were acquired during a breath hold from apical four-chamber and RV-focused views, respectively (16). Images were optimised to include the entire ventricular cavity within the highest possible frame rate scan volume. Volume measurements were performed semi-automatically using TomTec Arena (Dynamic HeartModel and 3D Auto RV, Tomtec Imaging Systems GmbH, Unterschleißheim, Germany). Ventricular endocardial borders were identified at end-diastole and end-systole and manually adjusted if necessary to calculate 3D EDV, ESV, and EF. In sinus rhythm, datasets were acquired using a minimum of four-beat full-volume mode. In AF, measurements in single-beat high-volume rate mode were averaged over three different datasets. LA volume was measured at end-systole using the biplane method (Figure 1).

### 2.5 Statistical analysis

Variables were assessed for normality using Q–Q plots and the Shapiro–Wilk test. Continuous variables were reported as mean ± standard deviation or median (interquartile range) and categorical variables as frequencies (%). Between-group differences were assessed using Student's *t*-test or the Mann–Whitney *U* test for continuous variables and the chi-squared or Fisher's exact test for categorical variables, as appropriate. The receiver operating characteristic (ROC) curve analysis assessed the ability of strain measurements to discriminate H-PH, and optimum strain cut-offs were obtained using the Youden index. Areas under the curve (AUCs) with corresponding confidence intervals (CIs) were compared with DeLong's test. Correlation and agreement between CT and TTE measurements were assessed using the Pearson correlation coefficient and Bland–Altman analysis, respectively. Intra- and inter-observer reproducibility of strain measures was assessed using the intra-class correlation coefficient (ICC). Statistical significance was defined as *p* < 0.05. All analyses were performed using R 4.4.0 (R Foundation for Statistical Computing, Vienna, Austria).

## 3 Results

### 3.1 Study population

Table 1 describes the clinical characteristics of 93 study patients, comprising 43 (46.2%) with H-PH and 50 (53.8%) with L-PH. Patients with H-PH had a higher prevalence of AF, elevated N-terminal pro-B-type natriuretic peptide (NT-proBNP) levels, more frequent New York Heart Association functional class ≥ III, shorter six-minute walk test (6MWT) distance, lower quality of life on the Kansas City Cardiomyopathy Questionnaire (KCCQ), and greater frailty (all *p* < 0.05). On TTE, patients with H-PH had a smaller AVA, lower LV-EF, reduced RV function, larger atria, higher LV filling pressures (E/e') and, as expected, greater TR Vmax (all *p* < 0.05). The total dose-length product (DLP) was significantly higher in H-PH than in L-PH [933 [774, 1,230] mGycm vs. 728 [606, 1,028] mGycm, *p* = 0.019]. There was no significant inter-group difference in heart rate at the time of CT acquisition.

**Table 1:** Baseline characteristics.

Characteristics	All patients (*n* = 93)	L-PH (*n* = 50)	H-PH (*n* = 43)	*p*-value
Age (years)	82.4 ± 5.6	81.5 ± 5.0	83.5 ± 6.1	0.100
Gender (female)	30 (32%)	19 (38%)	11 (26%)	0.201
BMI (kg/m^2^)	27.2 ± 5.9	26.7 ± 5.7	27.9 ± 6.2	0.332
Hypertension	74 (80%)	41 (82%)	33 (77%)	0.531
Hypercholesteremia	58 (62%)	33 (66%)	25 (58%)	0.435
Diabetes mellitus	34 (37%)	22 (44%)	12 (28%)	0.108
Current/ex-smoker	32 (34%)	20 (40%)	12 (28%)	0.221
AF/flutter	24 (26%)	2 (4.0%)	22 (51%)	<0.001*
NYHA ≥ III	44 (47%)	12 (24%)	32 (74%)	<0.001*
6MWT (m)	191 (122, 281)	239 (174, 304)	136 (96, 202)	<0.001*
CFS ≥4	43 (46%)	15 (30%)	28 (65%)	<0.001*
Katz <6	22 (24%)	6 (12%)	16 (37%)	0.004*
KCCQ	40 (27, 59)	49 (31, 70)	35 (23, 40)	0.002*
eGFR (mL/min/1.73m^2^)	63 ± 19	67 ± 17	58 ± 21	0.019*
NT-proBNP (ng/L)	1,107 (520, 2,331)	619 (429, 1,290)	1,978 (1,087, 4,040)	<0.001*
AVA (cm^2^)	0.65 ± 0.16	0.68 ± 0.17	0.61 ± 0.13	0.023*
Simpson's LV-EF (%)	62.3 ± 6.1	66.6 ± 4.5	57.2 ± 2.7	<0.001*
LV mass (g)	226 (190, 279)	217 (165, 265)	243 (199, 305)	0.018*
LA area (cm^2^)	27 (22, 32)	24 (19, 27)	30 (27, 36)	<0.001*
E/e'	17 (13, 20)	15 (11, 18)	18 (15, 22)	0.002*
PASP (mmHg)	36 (32, 64)	32 (26, 36)	64 (61, 75)	<0.001*
PV acceleration time (ms)	103 ± 22	120 ± 9	84 ± 16	<0.001*
TR Vmax (m/s)	2.80 (2.38, 3.38)	2.44 (2.23, 2.78)	3.39 (3.28, 3.78)	<0.001*
TAPSE (mm/mmHg)	20.4 ± 5.4	23.7 ± 3.6	16.6 ± 4.6	<0.001*
RV FAC (%)	40 ± 10	46 ± 6	32 ± 9	<0.001*
RV basal diameter (mm)	4.52 ± 0.74	4.06 ± 0.66	5.06 ± 0.37	<0.001*
RA area (cm^2^)	20 (16, 25)	16 (14, 18)	25 (22, 33)	<0.001*
IVC diameter (mm)	1.83 ± 0.72	1.19 ± 0.28	2.55 ± 0.22	<0.001*
Heart rate (bpm)	68.6 ± 6.89	68.1 ± 5.97	68.2 ± 7.66	0.324
Total DLP (mGycm)	856.9 (640, 1,137)	728 (606, 1,028)	933 (774, 1,230)	0.019*

AF, atrial fibrillation; AVA, aortic valve area; BMI, body mass index; CFS, clinical frailty score; DLP, dose-length product; eGFR, estimated glomerular filtration rate; ESV, end-systolic volume; FAC, fractional area change; H-PH, high probability of pulmonary hypertension; IQR, interquartile range; IVC, inferior vena cava; KCCQ, Kansas City cardiomyopathy questionnaire; LA, left atrium; L-PH, low probability of pulmonary hypertension; LV, left ventricle; LV-EF, left ventricular ejection fraction; NT-proBNP, N-terminal pro B-type natriuretic peptide; NYHA, New York Heart Association functional class; PASP, pulmonary artery systolic pressure; PV, pulmonary valve; RA, right atrium; TAPSE, tricuspid annular plane systolic excursion; RV, right ventricle; SD, standard deviation; TR, tricuspid regurgitation; 6MWT, six-minute walk test.

Data are reported as mean ± SD, median (IQR), or frequency (%). The *p*-value refers to the comparison between L-PH and H-PH groups.

* *p* < 0.05.

### 3.2 Agreement of CT and TTE

**Figure 2:**
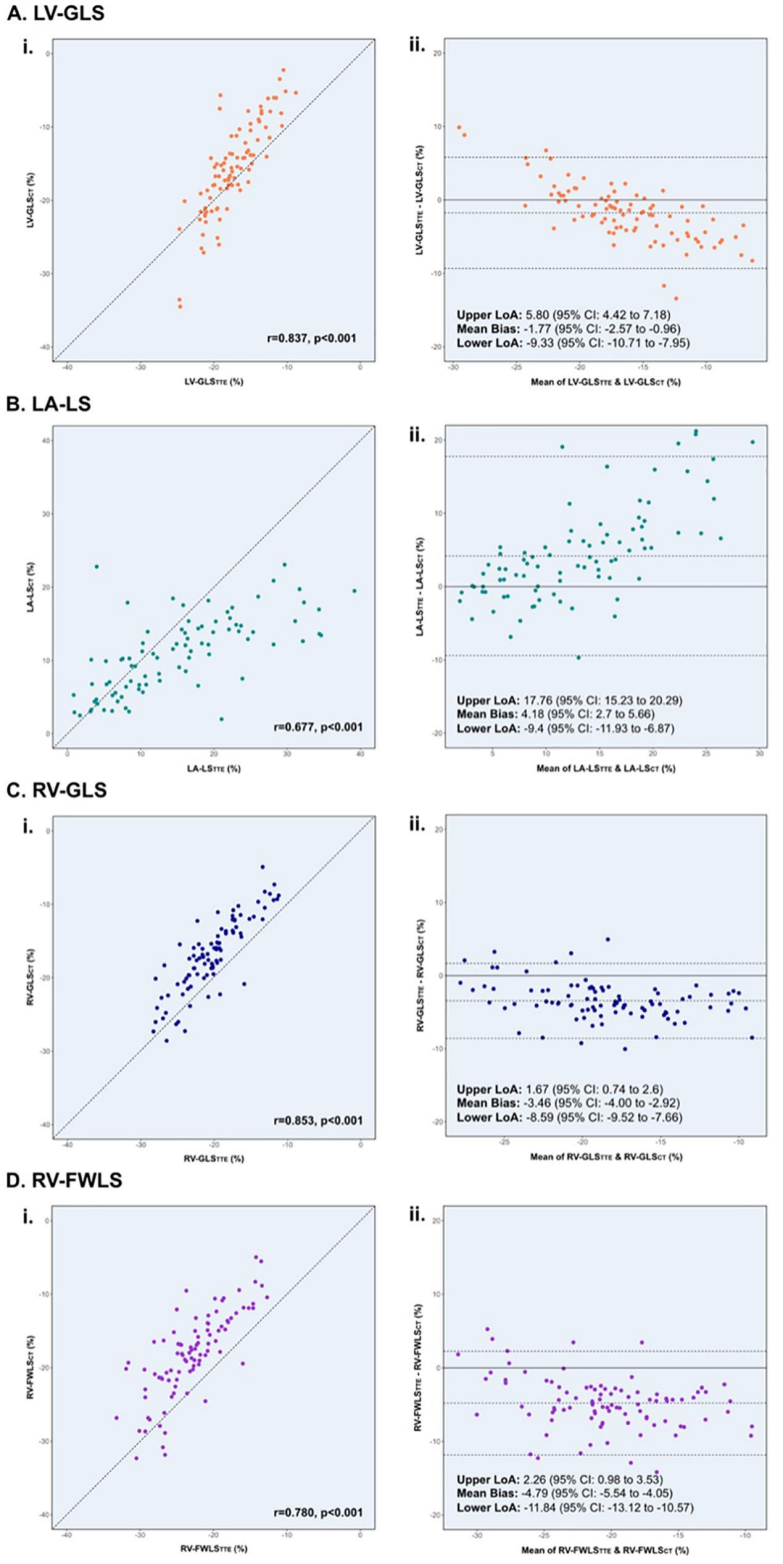
Pearson correlation coefficients **(i)** and Bland–Altman **(ii)** analyses for LV-GLS **(A)**, LA-LS **(B)**, RV-GLS **(C)**, and RV-FWLS **(D)** between 3D CT and 2D TTE. Inter-modality correlation coefficients (r) are reported alongside their corresponding *p*-values. The mean inter-modality difference (bias) and LoA are shown in the dashed lines. CI, confidence interval; CT, computed tomography; LA-LS, left atrial longitudinal reservoir strain; LoA, limit of agreement; LV-GLS, left ventricular global longitudinal strain; RV-GLS, right ventricular global longitudinal strain; RV-FWLS, right ventricular free-wall longitudinal strain; TTE, transthoracic echocardiography.

Table 2 presents paired comparisons of longitudinal strain, myocardial volumes, and EF measurements obtained using CT and TTE. CT-derived measures of LV-GLS, LA-LS, RV-GLS, and RV-FWLS were significantly lower in magnitude than the corresponding TTE values (all *p* < 0.001). Strong inter-modality correlations were observed for LV-GLS (r = 0.837), RV-GLS (r = 0.853), and RV-FWLS (r = 0.780), whereas the correlation for LA-LS was moderate (r = 0.677) (all *p* < 0.001) (Figure 2). Among all strain parameters, RV-FWLS showed the greatest under-estimation bias on CT compared with TTE [mean bias: −4.78%; 95% limit of agreement (LoA): 2.26 and −11.8%].

**Table 2:** Comparison of longitudinal strain, myocardial volume, and ejection fraction measurements using CT and TTE.

Parameter	CT (*n* = 93)	TTE (*n* = 93)	*p*-value
Strain			
LV-GLS (%)	−15.6 ± 6.3	−17.2 ± 3.6	0.029*
LA-LS (%)	11.0 ± 5.0	15.0 ± 9.0	<0.001*
RV-GLS (%)	−16.8 ± 5.0	−20.3 ± 4.1	<0.001*
RV-FWLS (%)	−18.0 ± 5.7	−22.8 ± 4.6	<0.001*
Volumes and EF			
LV-EDV (mL)	128 ± 25	116 ± 22	<0.001*
LV-ESV (mL)	50 ± 13	45 ± 10	0.013*
LV-EF (%)	59 (55, 67)	60 (57, 64)	0.933
LA-ESV (mL)	138 (112, 173)	90 (67, 113)	<0.001*
RV-EDV (mL)	156 (135, 196)	144 (123, 177)	0.012*
RV-ESV (mL)	64 (49, 107)	63 (46, 95)	0.438
RV-EF (%)	58 (45, 65)	56 (43, 63)	0.223

CT, computed tomography; EDV, end-diastolic volume; EF, ejection fraction; ESV, end-systolic volume; IQR, interquartile range; LA, left atrium; LA-LS, left atrial longitudinal reservoir strain; LV, left ventricle; LV-GLS, left ventricular global longitudinal strain; RV, right ventricle; RV-FWLS, right ventricular free-wall longitudinal strain; RV-GLS, right ventricular global longitudinal strain; SD, standard deviation; TTE, transthoracic echocardiography.

Data are reported as mean ± SD or median (IQR). The *p*-value refers to the comparison between TTE and CT measurements.

* *p* < 0.05.

With respect to myocardial volumes, TTE significantly underestimated LV-EDV, LV-ESV, LA-ESV, and RV-EDV compared with CT (all *p* < 0.05), while no significant differences were observed in LV-EF and RV-EF (*p* > 0.05). Strong inter-modality correlations were observed for LV-EF, LV-EDV, and LV-ESV (r≥0.920, *p* < 0.001), for RV-EF, RV-EDV, and RV-ESV (r≥0.950, *p* < 0.001 for all), and for LA-ESV (r = 0.855, *p* <0.001) (Supplementary Figures 1–S3). Among volumetric parameters, LA-ESV showed the greatest under-estimation bias on TTE compared with CT (mean bias: −50.7 mL; 95% LoA: 6.6 and −108.0 mL) (Supplementary Figure 3).

### 3.3 Association of myocardial strain with H-PH in AS

**Figure 3:**
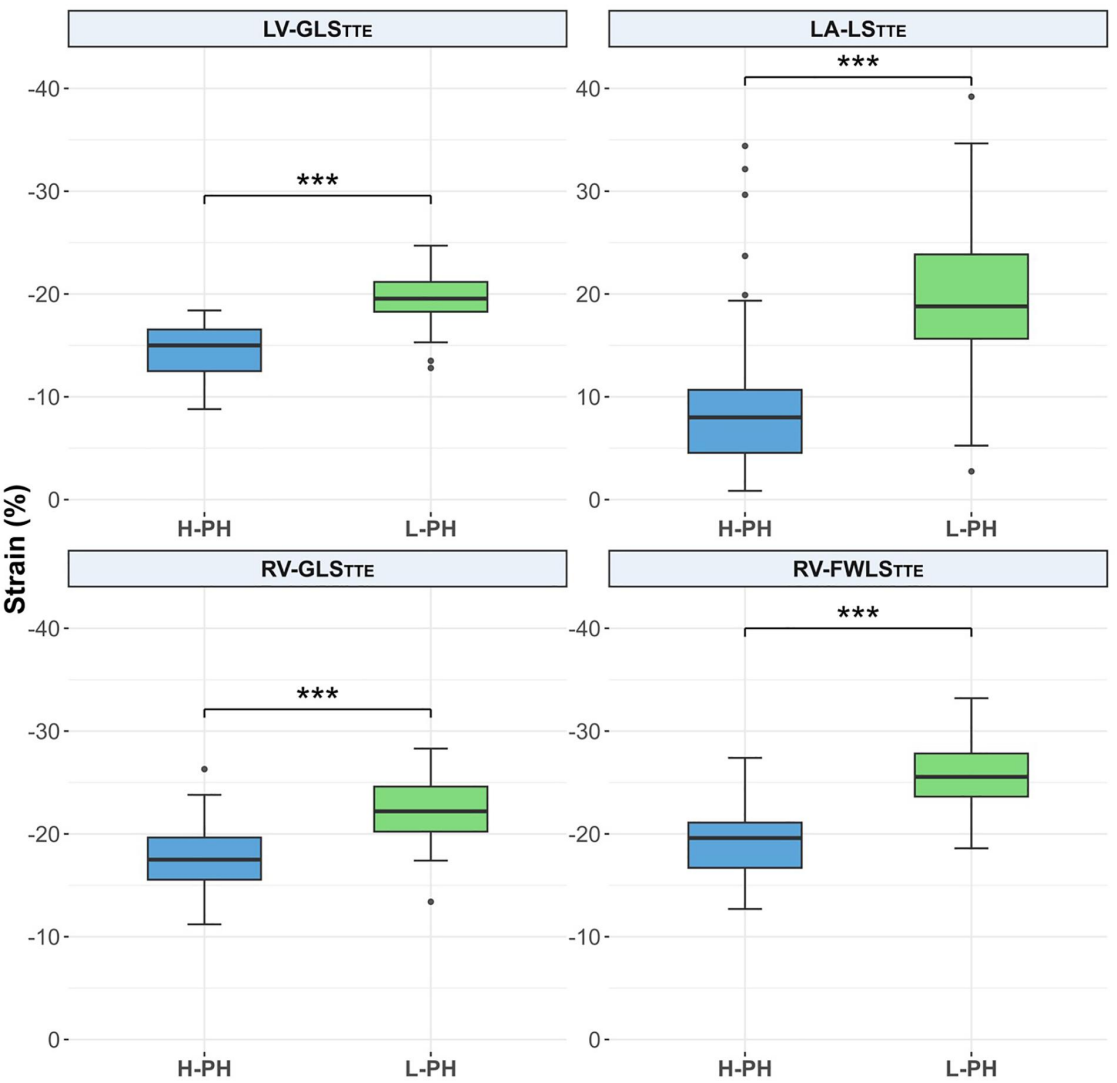
Comparison of 2D TTE-derived longitudinal strain parameters between patients with H-PH and L-PH. Box-and-whisker plots: the box length represents the IQR; the horizontal box line represents the median; the whiskers denote the maximum and minimum values excluding outliers (shown as dots); *** denotes *p* < 0.001. H-PH, high probability of pulmonary hypertension; IQR, interquartile range; LA-LS, left atrial longitudinal reservoir strain; L-PH, low probability of pulmonary hypertension; LV-GLS, left ventricular global longitudinal strain; RV-GLS, right ventricular global longitudinal strain; RV-FWLS, right ventricular free-wall longitudinal strain; TTE, transthoracic echocardiography; 2D, two-dimensional.

**Figure 4:**
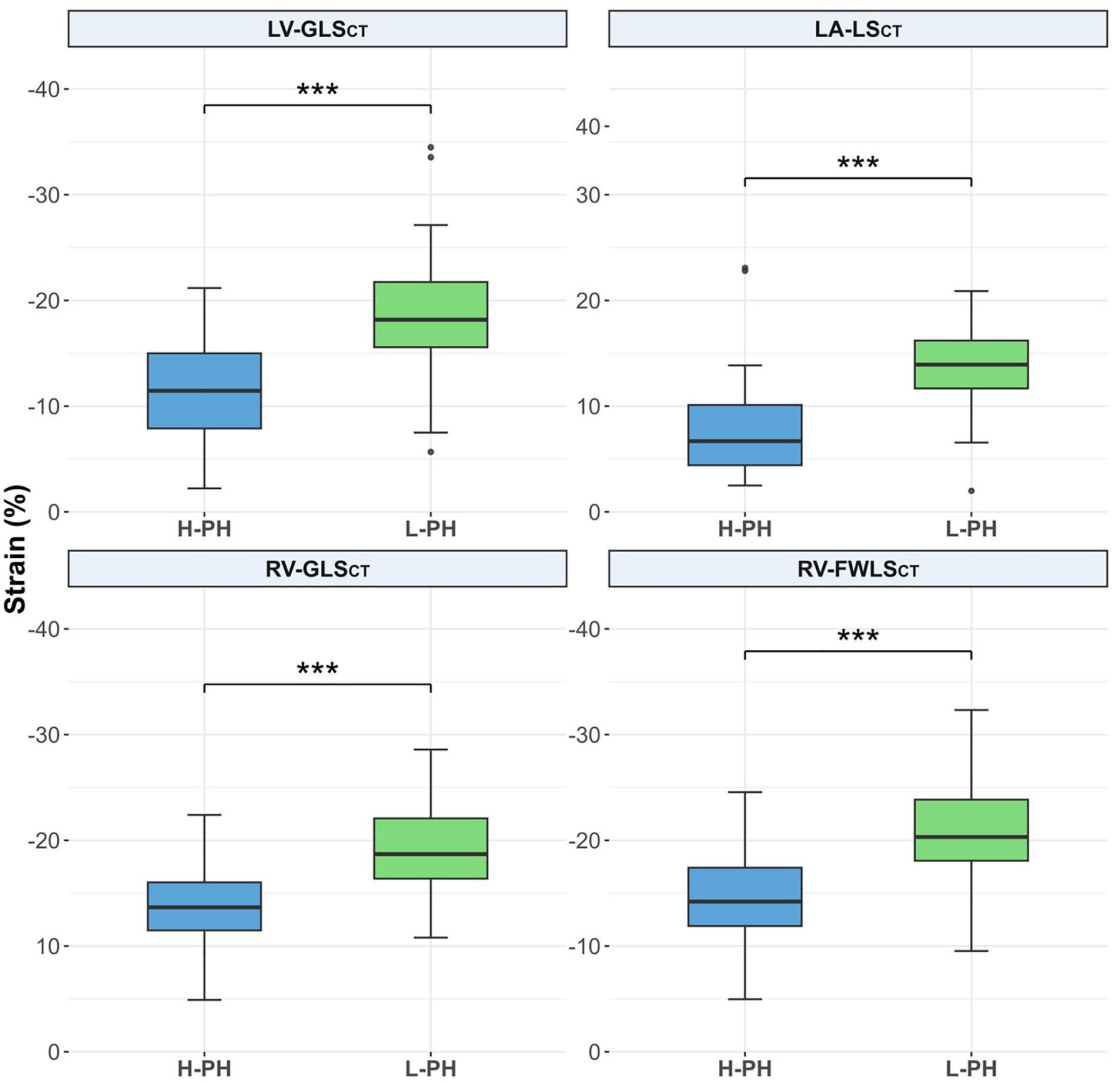
Comparison of 3D CT-derived longitudinal strain parameters between patients with H-PH and L-PH. Box-and-whisker plots: the box length represents the IQR; the horizontal box line represents the median; the whiskers denote the maximum and minimum values excluding outliers (shown as dots); *** denotes *p* < 0.001. CT, computed tomography; H-PH, high probability of pulmonary hypertension; IQR, interquartile range; LA-LS, left atrial longitudinal reservoir strain; L-PH, low probability of pulmonary hypertension; LV-GLS, left ventricular global longitudinal strain; RV-GLS, right ventricular global longitudinal strain; RV-FWLS, right ventricular free-wall longitudinal strain; 3D, three-dimensional.

Supplementary Tables 1, 2 present detailed comparisons of myocardial strain, volume, and EF measurements obtained using TTE and CT in AS patients with L-PH and H-PH. All longitudinal strain parameters on TTE were significantly reduced in H-PH compared with L-PH: LV-GLSTTE (−19.6 ± 2.4% vs. −14.5 ± 2.5%, *p* < 0.001), LA-LSTTE (20 ± 8% vs. 10 ± 8, *p* < 0.001), RV-GLSTTE (−22.5 ± 3.2% vs. −17.7 ± 3.6%, *p* < 0.001), and RF-FWLSTTE (−25.8 ± 3.1% vs. −19.3 ± 3.5%, *p* < 0.001) (Figure 3). Similarly, all CT-derived longitudinal strain values were significantly lower in H-PH than in L-PH: LV-GLSCT (−18.9 ± 5.6% vs. −11.5 ± 4.5%, <0.001), LA-LSCT (13.6 ± 3.8% vs. 7.7 ± 4.7%, *p* < 0.001), RV-GLSCT (−19.4 ± 4.2% vs. −13.7 ± 4.0%, *p* < 0.001), and RV-FWLSCT (−21.1 ± 5.1% vs. −14.4 ± 4.1%, *p* < 0.001) (Figure 4).

**Figure 5:**
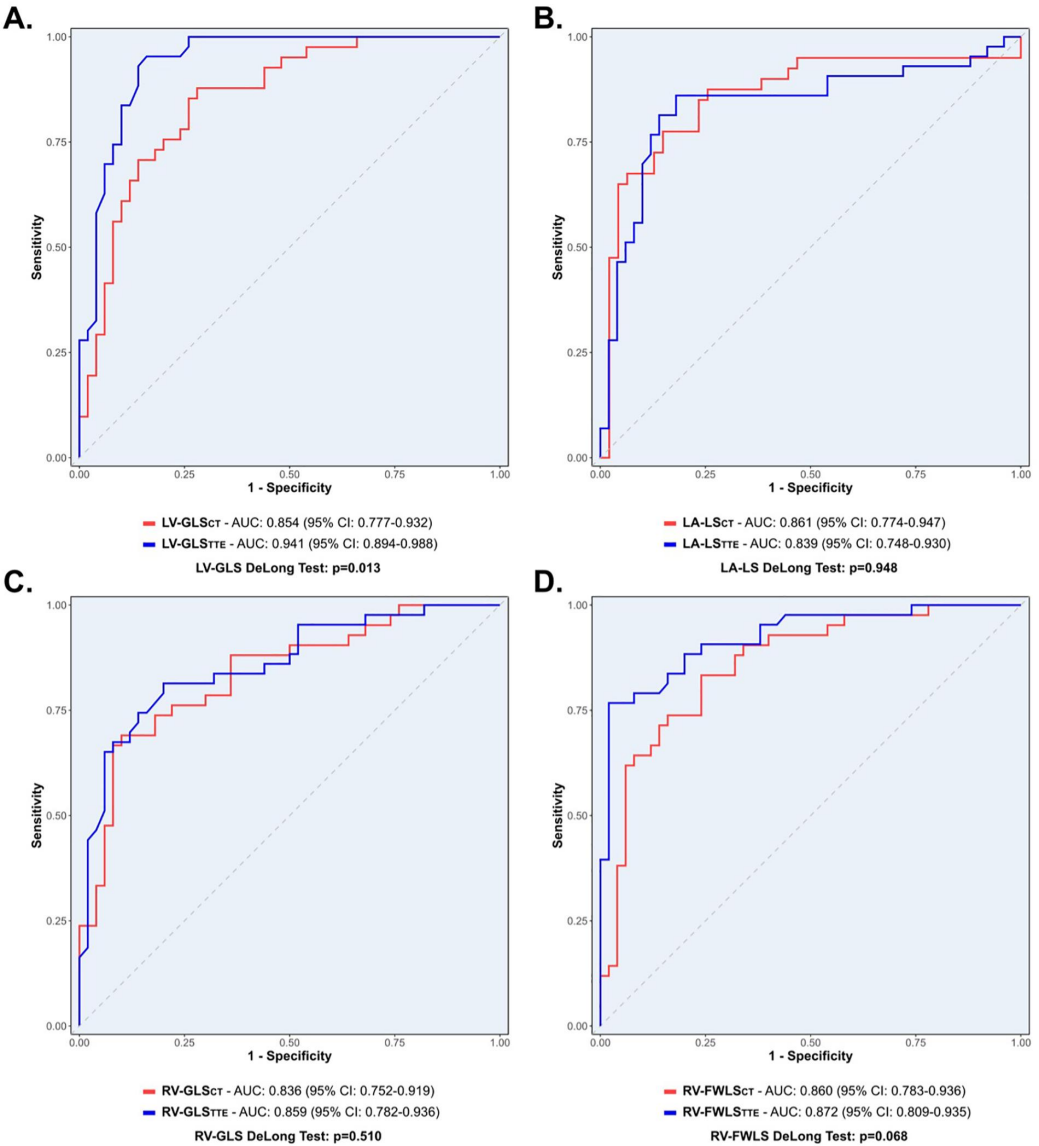
Receiver operating characteristic analyses for detecting H-PH using LV-GLS **(A)**, LA-LS **(B)**, RV-GLS **(C)**, and RV-FWLS **(D)** from 2D TTE (blue) and 3D CT (red). Receiver operating characteristic curve analyses are reported as the AUC with the corresponding 95% CIs and *p*-values. AUC, area under the curve; CI, confidence interval; CT, computed tomography; H-PH, high probability of pulmonary hypertension; LA-LS, left atrial longitudinal reservoir strain; LV-GLS, left ventricular global longitudinal strain; RV-GLS, right ventricular global longitudinal strain; RV-FWLS, right ventricular free-wall longitudinal strain; TTE, transthoracic echocardiography; 2D, two-dimensional; 3D, three-dimensional.

ROC analyses were performed to assess the discriminatory ability of TTE- and CT-derived longitudinal strain to detect H-PH in severe AS (Supplementary Table 3). In a head-to-head inter-modality comparison, it was found that LV-GLSTTE was superior to LV-GLSCT for detecting H-PH [AUC: 0.94 [95% CI 0.89–0.99] vs. 0.85 [95% CI 0.78–0.93], *p* = 0.013], whereas differences for LA-LS, RV-GLS, and RV-FWLS were non-significant (all *p* > 0.05) (Figure 5). The optimal longitudinal strain cut-off values for identifying H-PH were consistently lower in magnitude on CT than on TTE: LV-GLS (−16.4% vs. −17.9%), LA-LS (10.2% vs. 14.4%), RV-GLS (−15.3% vs. −20.1%), and RV-FWLS (−18.0% vs. −21.2%) (Table 3). In sub-analyses of volume-based measures, the associations of LV-EF, RV-EF, and LA-ESV with H-PH did not differ significantly between CT and TTE (all *p* < 0.05) (Supplementary Table 4).

**Table 3:** Receiver operating characteristic analysis of TTE- and CT-derived longitudinal strain to identify optimal cut-offs for the detection of H-PH.

Strain parameter	Cut-off (%)	Specificity (95% CI)	Sensitivity (95% CI)
2D TTE			
LV-GLS	−17.9	0.840 (0.740–0.940)	0.954 (0.884–1.000)
LA-LS	14.4	0.820 (0.700–0.920)	0.861 (0.767–0.954)
RV-GLS	−20.1	0.800 (0.680–0.900)	0.814 (0.698–0.930)
RV-FWLS	−21.2	0.732 (0.620–0.831)	0.977 (0.930–1.000)
3D CT			
LV-GLS	−16.4	0.720 (0.600–0.840)	0.878 (0.781–0.976)
LA-LS	10.2	0.851 (0.745–0.936)	0.775 (0.650–0.900)
RV-GLS	−15.3	0.900 (0.820–0.980)	0.691 (0.548–0.810)
RV-FWLS	−18.0	0.760 (0.640–0.880)	0.833 (0.714–0.929)

AUC, area under the curve; CI, confidence interval; CT, computed tomography; H-PH, high probability of pulmonary hypertension; LA-LS, left atrial longitudinal reservoir strain; LV-GLS, left ventricular global longitudinal strain; RV-GLS, right ventricular global longitudinal strain; RV-FWLS, right ventricular free-wall longitudinal strain; TTE, transthoracic echocardiography; ROC, receiver operating characteristic.

Optimal strain cut-off values were determined from ROC curves using the Youden index. Cut-off values were reported with their corresponding sensitivity, specificity, and 95% CIs.

### 3.4 Reproducibility of strain measurements

There was excellent inter- and intra-observer reproducibility (ICC > 0.9) for TTE- and CT-derived longitudinal strain measurements (Supplementary Table 5).

## 4 Discussion

This is the first study to compare multi-chamber 3D strain on CT with 2D strain on TTE and evaluate their association with PH in severe AS. The major findings are as follows: (1) 4D TAVI CT allows assessment of LV, LA, and RV motion tracking for 3D strain analysis with high reproducibility; (2) CT-derived 3D longitudinal strain is strongly correlated with 2D TTE-derived strain for the LV and RV and moderately so for the LA; (3) peak systolic longitudinal strain of the LV, LA, and RV is lower in AS patients with H-PH compared with those with L-PH; and (4) all longitudinal strain values (and the optimal cut-offs for identifying H-PH) are of lower magnitude on 3D CT than on 2D TTE, indicating modality-specific thresholds.

The prognostic value of CT-derived strain has been previously demonstrated in patients with severe AS using 2D cine loops reconstructed from 3D TAVI CT datasets. Using this simplified planar approach, baseline impairment in LV-GLS, RV-GLS, and LA-LS on CT has been independently associated with all-cause mortality following TAVI (17–19). However, myocardial deformation is inherently 3D and complex, reflecting the regional summation of forces generated by helically oriented myocardial fibres. Accordingly, CT-derived 3D strain assessment has been applied to the LV and shown to be independently associated with mortality and re-hospitalisation following TAVI (20). Building on this important work, the current study confirms the feasibility and reproducibility of 3D strain measurement on CT in patients undergoing TAVI evaluation and extends this approach to multi-parametric assessment of the LV, LA, and RV as components of whole heart extra-valvular modelling in AS. Because retrospective ECG-gated CT is routinely used for TAVI planning, the incorporation of multi-chamber 3D strain assessment broadens its functional utility without additional radiation exposure. This approach has the potential to enhance pre-procedural risk stratification and provide patient-specific evaluation of sub-clinical myocardial dysfunction, thereby improving the prediction of adverse outcomes. This concept is supported by prior work staging AS severity using multi-chamber TTE-derived strain, which provides incremental prognostic value beyond traditional functional parameters for the prediction of all-cause mortality and re-hospitalisation following TAVI (21). Wider applications may also include transcatheter mitral and tricuspid valve intervention cohorts, where routine 4D CT is required for pre-procedural evaluation.

Several previous studies have compared the agreement between 2D longitudinal strain measurements on CT and TTE in significant AS. These identified moderate-to-strong inter-modality correlations for LV-GLS (r = 0.60–0.82) (22, 23) and LA-LS (r = 0.66–0.87) (24, 25) but only limited evidence of a weak correlation for RV-GLS and RV-FWLS (r = 0.19–0.37) (23). Our study extends the existing literature by demonstrating strong correlations between 3D CT and 2D TTE for LV-GLS, RV-GLS, and RV-FWLS and a moderate correlation for LA-LS. Differences in strain correlations across myocardial chambers and studies may be explained by the extent of CT-derived volume over-estimation compared with TTE. The lower correlation for LA-LS in our study may be attributable to significantly higher LA-ESV estimates on CT compared with TTE, suggesting that full visualisation of the LA was not consistently achieved with TTE. In comparison, the over-estimation of LV and RV volumes on CT was numerically smaller (although still significant), suggesting that similar cavity visualisation may have contributed to the strong inter-modality correlation. The higher correlations for RV-GLS and RV-FWLS in our study may reflect: (1) use of the same TTE vendor; (2) same-day CT and TTE acquisition; (3) inclusion of high-quality images; and (4) measurement of 3D CT strain, which avoids inherent foreshortening in single-slice 2D CT analysis, as described previously (20). These findings support the use of CT-derived 3D strain as a complementary, reproducible alternative to TTE for multi-chamber strain assessment in patients with AS undergoing TAVI evaluation. This may be particularly useful in those with suboptimal TTE images and/or contraindications to cardiac magnetic resonance imaging. Furthermore, given the comparable correlation coefficients for LV and LA strain, our CT strain methodology appears to be non-inferior to other available tools. The systematic under-estimation of strain by CT compared with TTE is consistent with previous reports and a likely reflection of the lower temporal resolution of CT, as fewer frames reduce the chance of capturing peak deformation. Indeed, in the present study, CT-derived strain analysis was performed using R-R interval reconstruction at 5% increments, yielding 20 frames per cardiac cycle, which is consistently lower than the minimum temporal resolution of ≥60 frames/s achieved with TTE (although it should be noted that direct measures of effective temporal resolution on CT were not available for the present analysis).

The present study demonstrates the clinical application of CT-derived 3D strain by identifying significant impairment of LV, LA, and RV longitudinal strain in AS patients with concurrent PH. These results emphasise how a comprehensive strain analysis can refine AS phenotyping and improve our understanding of the functional abnormalities that underlie poor prognosis in higher risk patients. Our findings are consistent with the pathophysiology of PH in AS, which reflects advanced disease where LV and LA compensatory mechanisms are exhausted (26). Reduction in RV strain in this setting is not surprising, given that the RV has a thinner wall and higher compliance than the LV, resulting in a lower tolerance to elevated afterload (27). These findings align with those of previous studies identifying LV and LA longitudinal strain as independent predictors of PH in severe AS and highlight the potential of left heart strain to identify patients with AS at risk of developing PH, who may benefit from earlier interventions (28, 29). Importantly, we found that longitudinal strain thresholds for identifying PH in AS were not interchangeable between CT and TTE. Despite systematically lower cut-offs, CT-derived LA and RV longitudinal strain proved comparable to TTE in the identification of PH. By contrast, TTE-derived LV longitudinal strain outperformed CT, likely as a result of its superior temporal resolution. Taken together, these findings highlight the need for modality and 3D strain specific cut-offs for the detection of PH in AS. Furthermore, our data support the use of CT as a valuable adjunct to TTE for strain-based assessment of progressive extra-valvular remodelling in patients with AS, particularly in TAVI candidates who already undergo routine pre-procedural planning CT. From a clinical implementation perspective, we hypothesise that a combined approach integrating both TTE- and CT-derived parameters may enhance the assessment of extra-valvular remodelling. Nevertheless, future studies with larger sample sizes will be required to establish whether CT-derived strain provides incremental phenotyping of cardiopulmonary remodelling beyond TTE.

## 5 Limitations

Despite the novel findings of this study, several limitations should be acknowledged. First, the study exclusion criteria resulted in a relatively selected cohort, which may limit the applicability of the findings and derived strain cut-offs to broader TAVI populations with more heterogeneous disease phenotypes. Second, a comparison with 2D CT strain would enhance the validity of the findings. Third, limited clinical follow-up and a relatively small, selected study population precluded evaluation of the incremental prognostic value of CT-derived 3D strain, representing an important area for future research that should concurrently consider CT- and TTE-derived 2D strain. Fourth, a comparison of circumferential and radial strain measurements between CT and TTE is needed. Fifth, right heart catheterisation (RHC) was not performed to confirm PH and its mechanism. Although RHC is not routinely performed during TAVI work-up and TTE reflects guideline-based practice for non-invasive PH assessment in severe AS, TTE is inherently susceptible to misclassification, particularly when pulmonary pressures are estimated as absolute mmHg values, owing to inherent limitations related to Doppler assessment, TR severity, and loading conditions. For this reason, we elected *a priori* to utilise categorisation of PH probability to mitigate misclassification. Therefore, strain cut-off values derived from ROC analysis should be interpreted as thresholds for identifying a high echocardiographic probability of PH, rather than specific pulmonary pressure values. Sixth, although the administered DLP was comparable to other studies performing CT strain analysis (23), future work should focus on reducing radiation exposure. Seventh, to promote a wider clinical adoption of CT-derived myocardial strain, future studies should prospectively compare strain measurement time between techniques. For reference, although not formally quantified in the present study, our CT methodology required approximately 5 min per chamber per case on a computer with 12 central processing unit cores. Eighth, the modest sample size and specific selection criteria contributed to inter-group differences in myocardial volumes, EFs, and certain clinical characteristics, which limited the feasibility of multivariable analyses. Therefore, the important question of whether CT-derived strain provides independent predictive value beyond established biomarkers such as myocardial volumes and EF for detecting extra-valvular remodelling associated with PH in severe AS warrants further investigation. Larger studies involving patients across the spectrum of extra-valvular remodelling are needed to assess the utility of multi-chamber 3D CT strain as a risk-stratification tool before TAVI.

## 6 Conclusions

This study demonstrates the feasibility of measuring 3D longitudinal strain in the LV, LA, and RV using motion tracking on 4D TAVI CT, with high inter- and intra-observer reproducibilities. An inter-modality comparison with TTE showed strong correlations for LV-GLS, RV-GLS, and RV-FWLS and a moderate correlation for LA-LS. Patients with severe AS and H-PH had reduced longitudinal strain on both TTE and CT than those with L-PH. CT-derived LA-LS, RV-GLS, and RV-FWLS was non-inferior to TTE in identifying H-PH, while TTE-derived LV-GLS was superior. In summary, 4D TAVI CT is a promising tool for multi-chamber 3D strain assessment in AS, warranting further studies to evaluate its prognostic value as a novel multi-parametric imaging technique.

## Data Availability

The raw data supporting the conclusions of this article will be made available by the authors without undue reservation.
