# Supplementary material for: Multi-chamber three-dimensional myocardial strain assessment by computed tomography: a comparison with speckle tracking echocardiography and association with pulmonary hypertension in severe aortic stenosis
Source: Front Cardiovasc Med. 2026 May 13;13:1774091. doi: 10.3389/fcvm.2026.1774091 (PMC13212198; doi:10.3389/fcvm.2026.1774091)
Supplement: Supplementary file 1 [file Datasheet1.docx]

**Multi-chamber three-dimensional myocardial strain assessment by computed tomography: comparison with speckle tracking echocardiography and association with pulmonary hypertension in severe aortic stenosis.**

**Supplementary Materials**

**Contents**

**Supplementary Table 1 Page 3**

**Supplementary Table 2 Page 4**

**Supplementary Table 3 Page 5**

**Supplementary Table 4 Page 6**

**Supplementary Table 5 Page 7**

**Supplementary Figure 1 Page 8**

**Supplementary Figure 2 Page 9**

**Supplementary Figure 3 Page 10**

**Supplementary methodology for 3D myocardial strain assessment Page 11**

**References Page 23**

**Supplementary Table 1.** Comparison of TTE- and CT-derived longitudinal strain parameters, stratified by L-PH and H-PH.

| **Parameter** | **Overall**  **(n = 93)** | **L-PH**  **(n = 50)** | **H-PH**  **(n = 43)** | **p value** |
| --- | --- | --- | --- | --- |
| **2D TTE** |  |  |  |  |
| LV-GLS_TTE_ (%) | -17.2 ± 3.6 | -19.6 ± 2.4 | -14.5 ± 2.5 | **<0.001*** |
| LA-LS_TTE_ (%) | 15.0 ± 9.0 | 20.0 ± 8.0 | 10.0 ± 8.0 | **<0.001*** |
| RV-GLS_TTE_ (%) | -20.3 ± 4.1 | -22.5 ± 3.2 | -17.7 ± 3.6 | **<0.001*** |
| RV-FWLS_TTE_ (%) | -22.8 ± 4.6 | -25.8 ± 3.1 | -19.3 ± 3.5 | **<0.001*** |
| **3D CT** |  |  |  |  |
| LV-GLS_CT_ (%) | -15.6 ± 6.3 | -18.9 ± 5.6 | -11.5 ± 4.5 | **<0.001*** |
| LA-LS_CT_ (%) | 10.9 ± 5.2 | 13.6 ± 3.8 | 7.7 ± 4.7 | **<0.001*** |
| RV-GLS_CT_ (%) | -16.8 ± 5.0 | -19.4 ± 4.2 | -13.7 ± 4.0 | **<0.001*** |
| RV-FWLS_CT_ (%) | -18.0 ± 5.7 | -21.1 ± 5.1 | -14.4 ± 4.1 | **<0.001*** |

Data are reported as mean ± SD or median (IQR). P value refers to comparison between L-PH and H-PH groups (*p<0.05).

**Abbreviations.** CT: computed tomography; H-PH: high probability of pulmonary hypertension; IQR: interquartile range; L-PH: low probability of pulmonary hypertension; LA-LS: left atrial longitudinal reservoir strain; LV-GLS: left ventricular global longitudinal strain; RV-GLS: right ventricular global longitudinal strain; RV-FWLS: right ventricular free-wall longitudinal strain; TTE: transthoracic echocardiography.

**Supplementary Table 2.** Comparison of myocardial volumes and ejection fractions from TTE and CT, stratified by L-PH and H-PH.

| **Parameter** | **Overall**  **(n = 93)** | **L-PH**  **(n = 50)** | **H-PH**  **(n = 43)** | **p value** |
| --- | --- | --- | --- | --- |
| **TTE** |  |  |  |  |
| LV-EDV (mL) | 116 ± 22 | 119 ± 23 | 112 ± 20 | 0.119 |
| LV-ESV (mL) | 45 ± 10 | 42 ± 10 | 49 ± 10 | **<0.001*** |
| LV-EF (%) | 60.8 ± 5.7 | 64.9 ± 4.3 | 56.0 ± 2.4 | **<0.001*** |
| LA-ESV (mL) | 90 (67, 113) | 78 (59, 96) | 109 (86, 140) | **<0.001*** |
| RV-EDV (mL) | 144 (123, 177) | 126 (115, 143) | 182 (145, 201) | **<0.001*** |
| RV-ESV (mL) | 63 (46, 95) | 46 (42, 54) | 102 (79, 124) | **<0.001*** |
| RV-EF (%) | 56 (43, 63) | 62 (59, 65) | 42 (37, 47) | **<0.001*** |
| **CT** |  |  |  |  |
| LV-EDV (mL) | 128 ± 25 | 130 ± 27 | 126 ± 23 | 0.396 |
| LV-ESV (mL) | 50 ± 13 | 44 ± 12 | 57 ± 11 | **<0.001*** |
| LV-EF (%) | 59 (55, 67) | 66 (63, 70) | 55 (53, 57) | **<0.001*** |
| LA-ESV (mL) | 138 (112, 173) | 122 (109, 149) | 155 (134, 198) | **<0.001*** |
| RV-EDV (mL) | 156 (135, 196) | 139 (127, 154) | 197 (165, 229) | **<0.001*** |
| RV-ESV (mL) | 64 (49, 107) | 50 (43, 58) | 117 (88, 132) | **<0.001*** |
| RV-EF (%) | 58 (45, 65) | 65 (61, 68) | 43 (37, 48) | **<0.001*** |

Data are reported as mean ± SD or median (IQR). P value refers to comparison between L-PH and H-PH groups (*p<0.05).

**Abbreviations**. CT: computed tomography; EDV: end-diastolic volume; EF: ejection fraction; ESV: end-systolic volume; IQR: interquartile range; LA: left atrium; LV: left ventricle; RV: right ventricle; TTE: transthoracic echocardiography.

**Supplementary Table 3.** Receiver operating characteristic analyses for detecting H-PH using TTE- and CT-derived longitudinal strain parameters.

| **Strain Parameter** | **AUC** | **95% CI** |
| --- | --- | --- |
| **2D TTE** |  |  |
| LV-GLS_TTE_ | 0.9412 | 0.8941-0.9882 |
| LA-LS_TTE_ | 0.8388 | 0.7482-0.9295 |
| RV-GLS_TTE_ | 0.8588 | 0.7819-0.9358 |
| RV-FWLS_TTE_ | 0.8716 | 0.8086-0.9346 |
| **3D CT** |  |  |
| LV-GLS_CT_ | 0.8541 | 0.7767-0.9316 |
| LA-LS_CT_ | 0.8606 | 0.7739-0.9474 |
| RV-GLS_CT_ | 0.8357 | 0.7522-0.9192 |
| RV-FWLS_CT_ | 0.8595 | 0.7827-0.9363 |

Receiver operating characteristic analyses are reported as AUC with the corresponding 95% CIs.

**Abbreviations.** AUC: area under the curve; CI: confidence interval; CT: computed tomography; H-PH: high probability of pulmonary hypertension; LA-LS: left atrial longitudinal reservoir strain; LV-GLS: left ventricular global longitudinal strain; RV-GLS: right ventricular global longitudinal strain; RV-FWLS: right ventricular free-wall longitudinal strain; TTE: transthoracic echocardiography.

**Supplementary Table 4.** Receiver operating characteristic curve analysis comparing CT and TTE for the discrimination of H-PH versus L-PH for LV-EF, RV-EF and LA-ESV.

| **Parameter** | **AUC (95% CI), TTE** | **AUC (95% CI), CT** | **p-value** |
| --- | --- | --- | --- |
| LV-EF | 0.989 (0.975-1.00) | 0.987 (0.970-1.00) | 0.827 |
| LA-ESV | 0.751 (0.642-0.844) | 0.724 (0.614-0.825) | 0.474 |
| RV-EF | 0.984 (0.967-1.00) | 0.982 (0.962-1.00) | 0.670 |

**Abbreviations.** AUC: area under the curve; CI: confidence interval; CT: computed tomography; H-PH: high probability of pulmonary hypertension; LA-ESV: left atrial end-systolic volume; LV-EF: left ventricular ejection fraction; RV-EF: right ventricular ejection fraction; TTE: transthoracic echocardiography.

**Supplementary Table 5.** Reproducibility assessment of TTE and CT longitudinal strain measurements.

|  | **Inter-observer variability**  **(n = 10)** | | | **Intra-observer variability**  **(n = 10)** | | |
| --- | --- | --- | --- | --- | --- | --- |
| **Strain Parameter** | **Mean difference ± SD** | **ICC**  **(95% CI)** | **p value** | **Mean difference ± SD** | **ICC**  **(95% CI)** | **p value** |
| **2D TTE** |  |  |  |  |  |  |
| LV-GLS_TTE_ | 1.13 ± 0.76 | 0.982  (0.502-0.997) | **<0.001*** | -0.15 ± 1.33 | 0.980  (0.920-0.995) | **<0.001*** |
| LA-LS_TTE_ | 0.69 ± 1.59 | 0.985  (0.940-0.996) | **<0.001*** | -0.09 ± 1.86 | 0.981  (0.921-0.995) | **<0.001*** |
| RV-GLS_TTE_ | -0.40 ± 1.50 | 0.975  (0.906-0.994) | **<0.001*** | -0.55 ± 1.63 | 0.969  (0.885-0.992) | **<0.001*** |
| RV-FWLS_TTE_ | -0.60 ± 1.39 | 0.980  (0.922-0.995) | **<0.001*** | 0.08 ± 1.53 | 0.980  (0.918-0.995) | **<0.001*** |
| **3D CT** |  |  |  |  |  |  |
| LV-GLS_CT_ | -0.12 ± 1.24 | 0.996  (0.985-0.999) | **<0.001*** | 0.03 ± 2.02 | 0.986  (0.944-0.997) | **<0.001*** |
| LA-LS_CT_ | 0.53 ± 1.12 | 0.987  (0.950-0.997) | **<0.001*** | 0.91 ± 1.75 | 0.964  (0.852-0.991) | **<0.001*** |
| RV-GLS_CT_ | -0.95 ±1.31 | 0.979  (0.882-0.995) | **<0.001*** | 0.62 ± 1.59 | 0.971  (0.888-0.993) | **<0.001*** |
| RV-FWLS_CT_ | -1.14 ± 1.98 | 0.972  (0.871-0.993) | **<0.001*** | 1.40 ± 2.89 | 0.915  (0.671-0.979) | **<0.001*** |

Data are reported and mean difference ± SD and ICC, with corresponding 95% CIs and p values.

**Abbreviations.** CI: confidence interval; CT: computed tomography; ICC: intraclass correlation coefficient; LA-LS: left atrial longitudinal reservoir strain; LV-GLS: left ventricular global longitudinal strain; RV-GLS: right ventricular global longitudinal strain; RV-FWLS: right ventricular free-wall longitudinal strain; SD: standard deviation; TTE: transthoracic echocardiography.

**Supplementary Figure 1.** Pearson correlation coefficients (i) and Bland–Altman (ii) analyses for LV-EF (A), LV-EDV (B), LV-ESV (C) between 3D CT and 3D TTE.


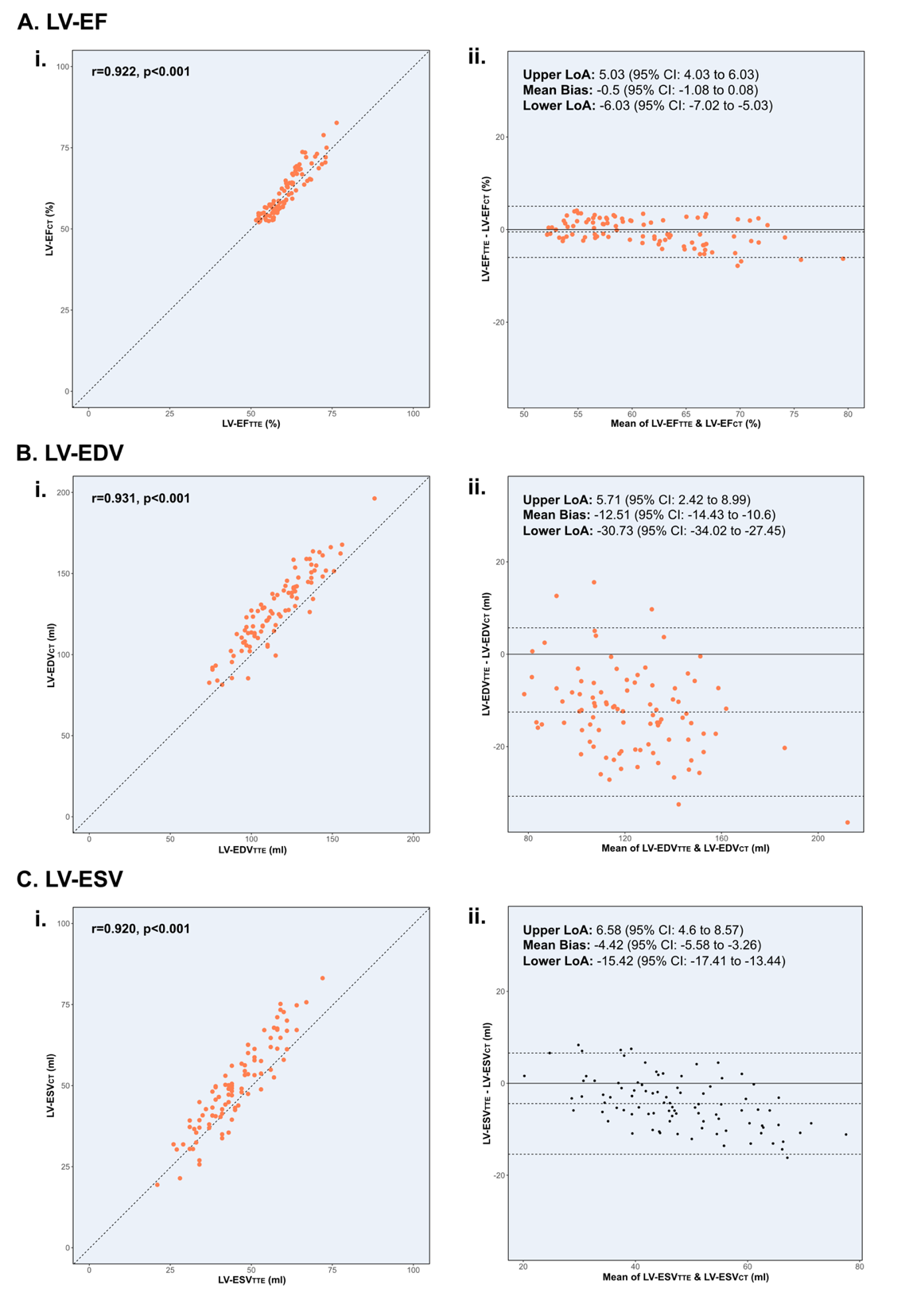


Inter-modality correlation coefficients (r) are reported alongside their corresponding p values. The mean inter-modality difference (bias) and LoA are shown in the dashed lines.

**Abbreviations.** CI: confidence interval; CT: computed tomography; EDV: end-diastolic volume; EF: ejection fraction; ESV: end-systolic volume; LoA: limit of agreement; LV: left ventricle; TTE: transthoracic echocardiography.

**Supplementary Figure 2.** Pearson correlation coefficients (i) and Bland–Altman (ii) analysis for RV-EF (A), RV-EDV (B), RV-ESV (C) between 3D CT and 3D TTE.


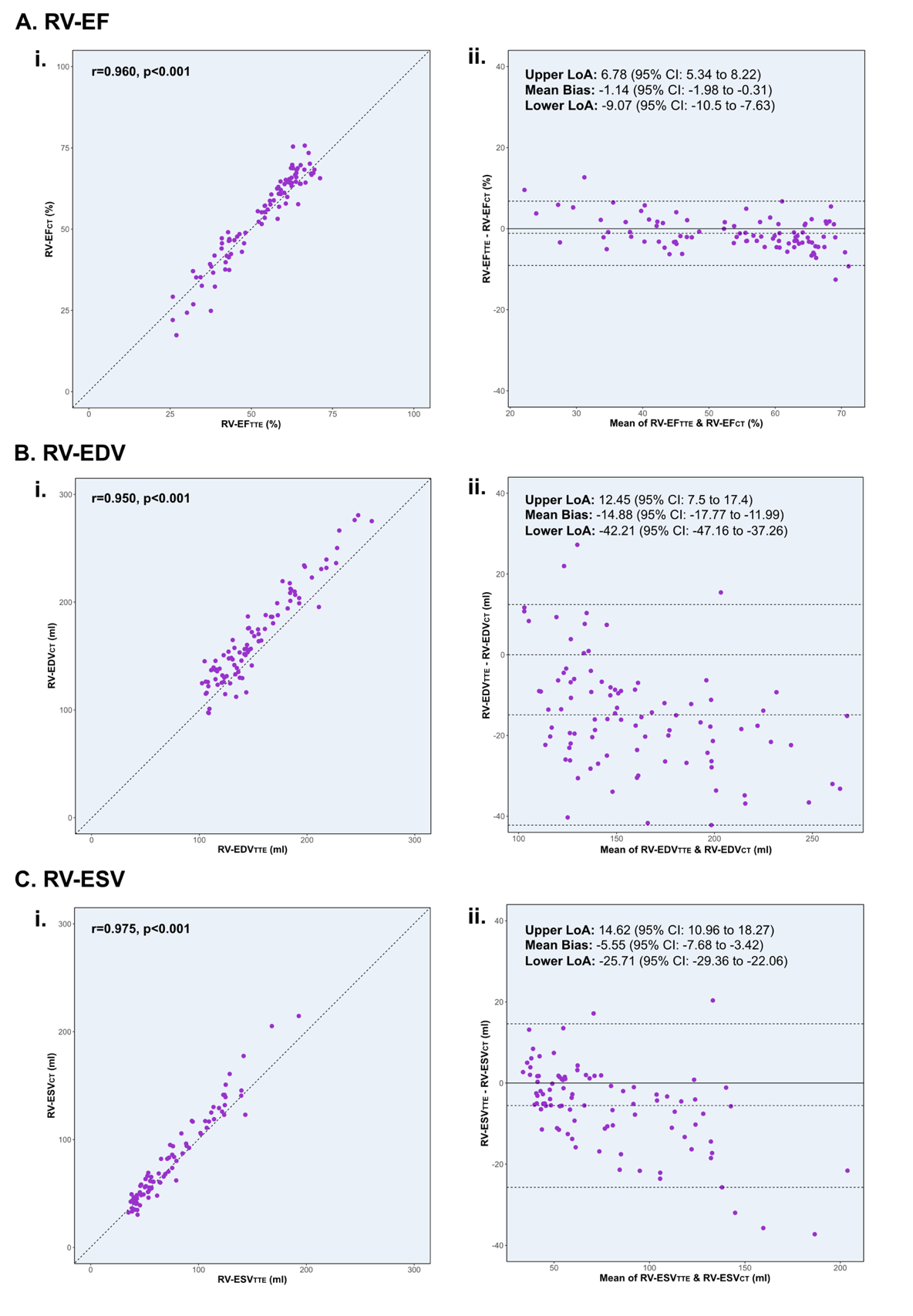


Inter-modality correlation coefficients (r) are reported alongside their corresponding p values. The mean inter-modality difference (bias) and LoA are shown in the dashed lines.

**Abbreviations.** CI: confidence interval; CT: computed tomography; EDV: end-diastolic volume; EF: ejection fraction; ESV: end-systolic volume; LoA: limit of agreement; RV: right ventricle; TTE: transthoracic echocardiography.

**Supplementary Figure 3.** Pearson correlation coefficient (i) and Bland–Altman (ii) analysis for LA-ESV between 3D CT and 2D TTE.


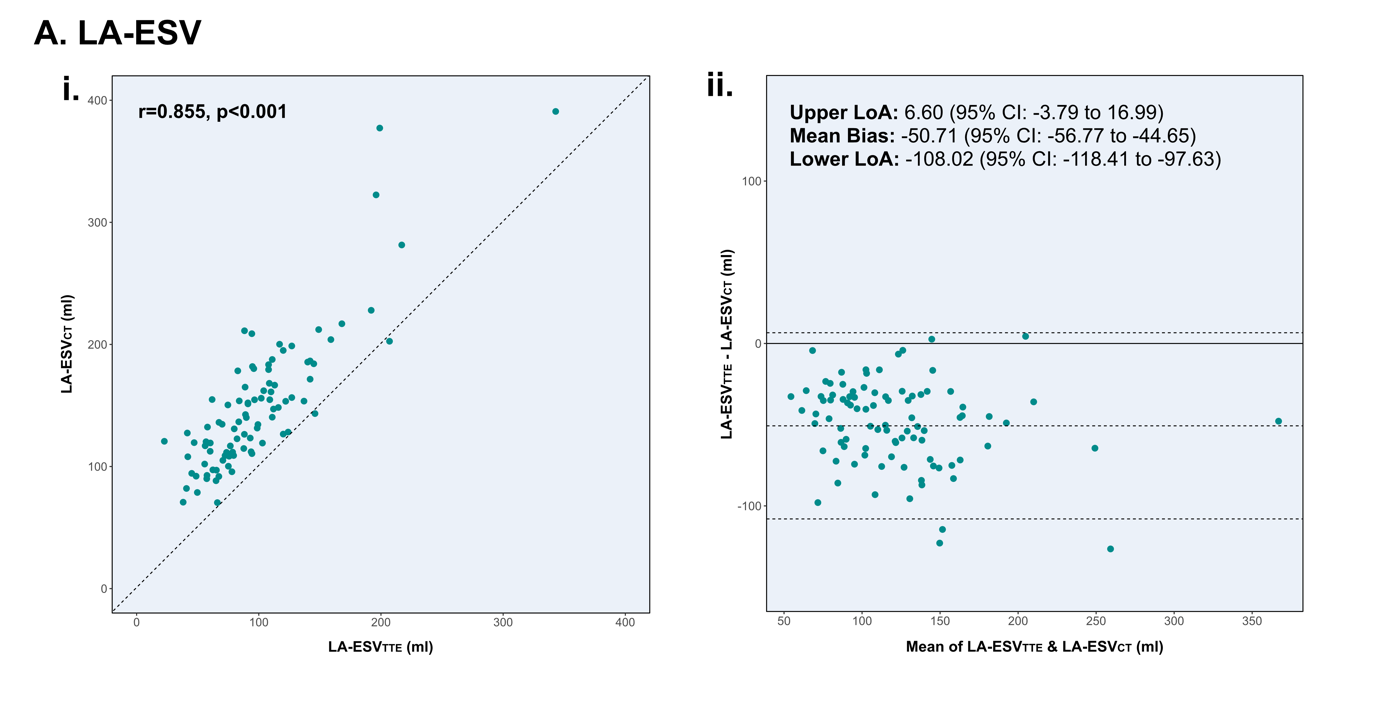


Inter-modality correlation coefficients (r) are reported alongside their corresponding p values. The mean inter-modality difference (bias) and LoA are shown in the dashed lines.

**Abbreviations.** CI: confidence interval; CT: computed tomography; ESV: end-systolic volume; LA: left atrium; LoA: limit of agreement; TTE: transthoracic echocardiography.

**1. Supplementary methodology for 3D myocardial strain assessment using cardiac computed tomography angiography**

Conventional assessment of 2-dimensional (2D) myocardial strain using speckle tracking transthoracic echocardiography (TTE) strongly depends on good ultrasound windows and high image quality, and has inherent limitations related to through-plane motion during a cardiac cycle and geometric assumptions.(1) Therefore, measurement of 3-dimensional (3D) cardiac strain offers a theoretical advantage in providing a more comprehensive description of complex myocardial motion. Retrospectively electrocardiogram (ECG)-gated cardiac computed tomography angiography (CT) is the gold standard imaging modality for the planning of transcatheter aortic valve implantation (TAVI) in severe aortic stenosis (AS). TAVI CT can acquire high-fidelity 4-dimensional (4D: 3D + time) images of the heart across the entire cardiac cycle, which can be used to evaluate 3D myocardial motion and calculate strain. This extends the utility of TAVI CT studies by providing additional functional information, which is currently not routinely measured and may improve patient risk-stratification. The following sections outline the methodological framework for myocardial strain quantification based on cardiac motion analysis with TAVI CT. We describe the previously validated workflows for measuring 3D left ventricular (LV) and left atrial (LA) strain on multiphase CT and demonstrate how this method was applied to track 3D right ventricular (RV) motion and calculate RV strain.

**2. Evaluation of myocardial motion using image registration with temporal sparse free form deformation**

Image registration refers to the spatial correspondence between two or more images. Registration is performed by computing displacement fields using motion tracking to align points in the initial image with their corresponding positions in subsequent images. Image registration is achieved if homologous points appear in the same spatial location in sequential images. Myocardial motion analysis is a specific application of image registration, whereby a sequence of image frames over the cardiac cycle are registered to map homologous points over time. This allows the displacement fields to track anatomical cardiac changes over time to quantify motion. Image registration for the assessment of cardiac motion using dynamic CT is analogous to the tracking of speckles in TTE. Temporal sparse free form deformation (TSFFD) is a validated deformable image registration technique for evaluating myocardial motion using dynamic CT imaging. It builds upon the widely used free form deformation approach by incorporating additional control point (CP) sparsity and temporal cyclicity constraints to model physiologically realistic cardiac deformation.(2,3) Motion tracking with TSFFD utilises all 20 CT frames acquired at 5% increments of the RR interval, which are spatially cropped to the target cardiac chamber as the input. TSFFD enables simultaneous group-wise registration of all subsequent frames to the reference image at end-diastole, allowing 3D myocardial motion to be tracked throughout the cardiac cycle using B-spline parametrisation to model spatially and temporally smooth deformations.(4) This approach is well-suited for evaluating cardiac motion, which is inherently periodic and temporally smooth over the cardiac cycle. The displacement fields generated from motion tracking can then be applied to deform individual cardiac chamber meshes from end-diastole to all subsequent frames in the cardiac cycle, enabling calculation of peak systolic strain. The TSFFD technique has been widely used for cardiac motion tracking on CT and has been validated for assessing LV and LA motion, as well as peak systolic strain.(2,5,6)

**3. Measurement of longitudinal myocardial strain based on motion tracking**

Peak systolic 3D strain is calculated from TSFFD-based motion tracking on CT using endocardial meshes of individual myocardial chambers. Longitudinal strain is quantified from systolic lengthening/shortening of the 3D myocardial mesh surface along pre-defined longitudinal axes and tangential to the endocardial surface. This section outlines the equations used to calculate peak systolic longitudinal myocardial strain relative to end-diastole (t=0%), which corresponds to the R-wave on the ECG.(5,6)

Peak-systolic 3D longitudinal strain describes both the magnitude and the direction of myocardial deformation on the endocardial surface relative to end-diastole. Longitudinal strain was defined as the change in length of myocardial surface models parallel to the longitudinal axis of the individual chambers, which were determined by manually selected landmarks. Longitudinal strain was calculated from the Green strain tensor, *E*, which was derived from the deformation gradient, *F*, for each element on the endocardial surface mesh using the formula:

$$E=\frac{1}{2}{(F}^{T}F-1)$$

The Green strain ($\epsilon_{G}$) was calculated for each strain parameter by projecting the Green strain tensor onto the corresponding longitudinal unit vector, which defined the direction of deformation:

$$\epsilon_{G}=\hat{N}^{T}E\hat{N}$$

where $\hat{N}$ represents the unit vector in the longitudinal direction on the endocardial surface mesh. The Green strain was then converted to linear myocardial strain ($\epsilon$) to express strain as a percentage:

$$\epsilon=\left( \sqrt{2\epsilon_{G}+1}-1 \right) \times100\%$$

**4. Workflow for measuring 3D LV global longitudinal strain**

This section describes the previously validated methodology of measuring 3D LV global longitudinal strain (LV-GLS) from LV motion tracking using the TSFFD method.(5) The workflow for generating patient-specific LV endocardial models, tracking LV motion and calculating strains from LV motion is described.

**4.1 Patient-specific LV model creation: segmentation and mesh**

Assessment of LV-GLS required the creation of endocardial meshes from end-diastolic segmentations of the LV. Patient-specific models of the LV endocardial surface were created using a convolutional neural network (CNN), which generated 3D whole-heart multilabel segmentations from CT images at end-diastole (t=0%) (Figure 1A).(7) The body of the LV was extracted from the multi-label segmentations using the LV blood pool label (Figure 1B). In cases where the CNN failed to produce an output, LV segmentations were created using a semi-automatic region growing tool and 3D interpolation in CemrgApp.(8) All segmentations were manually checked and corrected if necessary using CemrgApp. Endocardial surface meshes of the LV were generated from the LV segmentations using the Medical Image Registration Toolkit (MIRTK, <http://mirtk.github.io/>) (Figure 1C). To define the LV longitudinal axis, manual landmarks were selected in the 4-chamber, 2-chamber and short-axis views of the end-diastolic CT image: one on the LV apex and three on the mitral valve (MV) annular plane to create the MV annulus centre of mass (MV CoM) (Figure 2).

**Figure 1.**  LV segmentation and endocardial surface mesh creation from end-diastolic CT image.


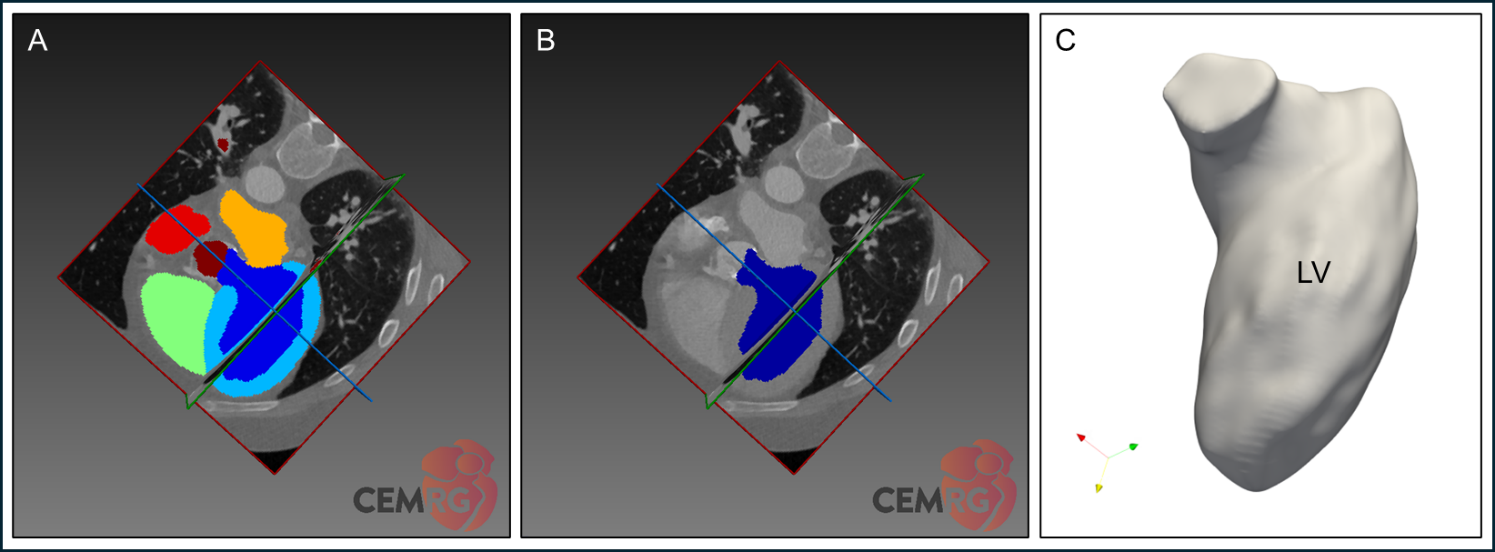


A) Multi-label segmentation output from the CNN; B) Segmentation of LV body from the LV blood pool label; C) 3D LV surface mesh extracted from LV segmentation.

**Abbreviations**. CT: cardiac computed tomography angiography; CNN: convolutional neural network; LV: left ventricle; 3D: 3-dimentional.

**Figure 2.** Manual landmark selection for defining LV longitudinal axis.


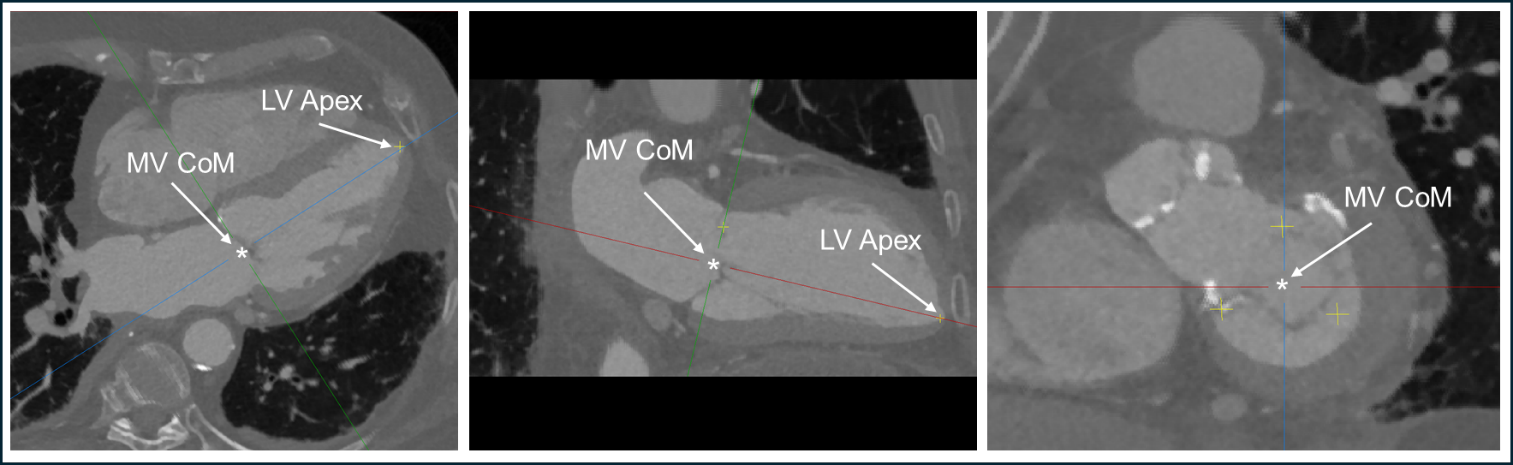


Landmarks were selected (green crosses) using the end-diastolic CT image in 4-chamber, 2-chamber and short-axis views to define the LV longitudinal axis between the MV CoM and LV apex.

**Abbreviations**. CoM: centre of mass; MV: mitral valve; LV: left ventricle.

**4.2 LV motion tracking**

The TSFFD method has been previously validated for LV motion tracking on retrospectively ECG-gated CT by optimising the two key TSFFD hyperparameters (bending energy [BE] and sparsity weight [SW]).(5) Razeghi *et al.*(5) found the optimal hyperparameter combination (BE, SW) for LV tracking by comparing tracked versus expert-selected landmarks on the LV at all phases of the cardiac cycle. The optimised TSFFD parameter configuration was then applied to accurately measure 3D LV-GLS in heart failure (HF) patients and control subjects. The authors identified increased LV dessynchrony and reduced LV-GLS from LV strain curves derived from 3D LV motion tracking in the HF patients compared to controls. We applied the same optimised TSFFD parameters to evaluate 3D LV motion and measure LV-GLS in our cohort of severe symptomatic AS patients using TAVI CT. The accuracy of motion tracking was checked by visual inspection of the tracked contours over the cardiac cycle.

**4.3 LV-GLS measurement**

Peak systolic 3D LV-GLS was quantified using the mean strain of all elements over the LV endocardial surface along the longitudinal axis, relative to end-diastole.(5) Figure 3 shows the LV endocardial mesh with the direction of longitudinal strain assessment.

**Figure 3.** Longitudinal direction on the LV endocardial surface.

Longitudinal direction for strain assessment on the LV endocardial surface.

**Abbreviations.** LV: left ventricle.

**5. Workflow for measuring 3D LA global longitudinal reservoir strain**

The method for tracking LA motion using TSFFD and measuring LA global longitudinal reservoir strain (LA-LS) was previously validated with retrospectively ECG-gated CT.(6) The workflow for generating patient-specific LA endocardial models, tracking motion and calculating LA-LS strain is described.

**5.1 Patient-specific LA model creation: segmentation and mesh**

Assessment of LA-LS required the creation of endocardial meshes from end-diastolic LA segmentations. Patient-specific models of the LA endocardium were generated from 3D whole-heart multilabel segmentations of end-diastolic CT images using a CNN (Figure 4A).(7) LA segmentations were extracted, visually inspected for accuracy and manually corrected if necessary using CemrgApp (Figure 4B).(8) In cases where the CNN failed to generate an output, LA segmentations were created using a semi-automatic region-growing and 3D interpolation tools in CemrgApp. LA endocardial surface meshes were generated from the LA segmentations using MIRTK (<http://mirtk.github.io/>). LA meshes were post-processed in CemrgApp by labelling and clipping the pulmonary veins and left-atrial-appendage extents, ensuring that only the LA body contributed to the strain calculation (Figure 4C).(9) Manual landmarks were selected to define the LV longitudinal axis as described previously (Figure 2), which was then extended to LA surface to create LA longitudinal axis.

**Figure 4.** LA segmentation and endocardial surface mesh creation from end-diastolic CT image.


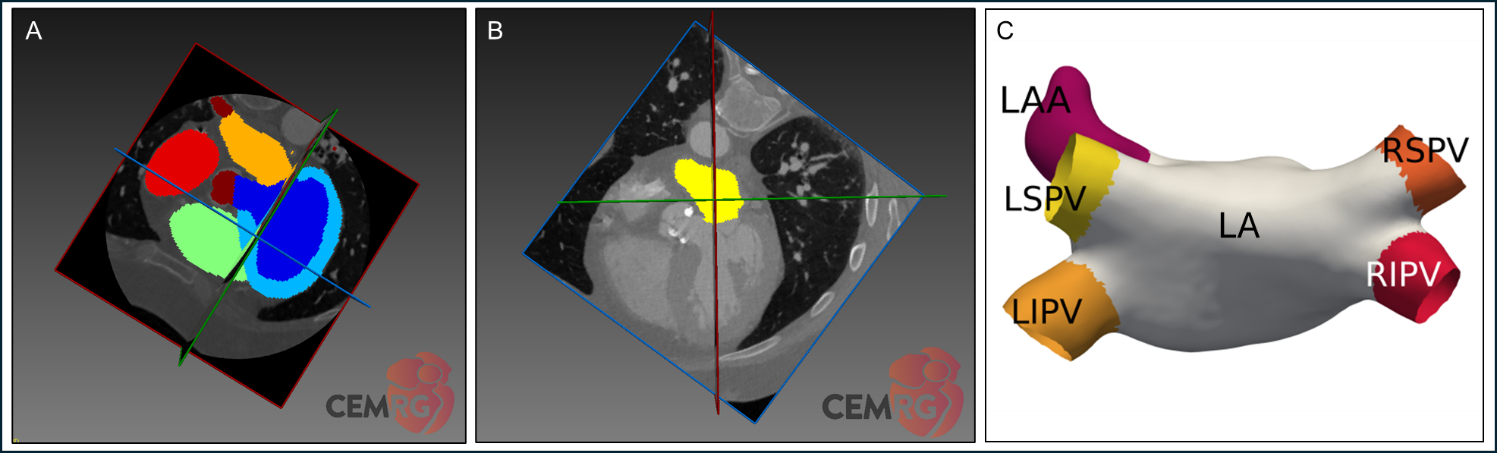


A) Multi-label myocardial segmentation derived from the CNN; B) Segmentation of LA from the LA blood pool label; C) 3D LA surface meshes were extracted and post-processed, which included clipping of the labelled pulmonary veins and left atrial appendage (orange and red).

**Abbreviations**. CT: cardiac computed tomography angiography; CNN: convolutional neural network; LA: left atrium; LAA: left atrial appendage; LIPV: left inferior pulmonary vein; LSPV: left superior pulmonary vein; RIPV: right inferior pulmonary vein; RSPV: right superior pulmonary vein; 3D: 3-dimentional.

**5.2 LA motion tracking**

The TSFFD method was previously optimised specifically for tracking LA motion on CT.(6) Sillett *et al*.(6) established LA chamber-specific TSFFD optimisation using a systematic grid search through multiple combinations of two key TSFFD hyperparameters (SW, BE), which were applied to track the change in LA shape from end-diastole (t=0%) to end-systole (t=40%). The tracked LA anatomy was then compared with the corresponding anatomy created directly from the surface meshes and segmentations of the LA in end-systole (t=40%), which was treated as the ground truth. Motion tracking was considered accurate if the tracked and ground truth anatomies in end-systole were similar. The accuracy of motion tracking was evaluated using three error metrics that quantify differences between tracked and ground truth surface meshes and segmentations: average surface distance (ASD), directed Hausdorff distance (DHD) and Dice score coefficient (DSC).(10) The ASD is defined as the mean normal distance between the tracked and ground truth meshes in end-systole. The DHD is the maximal minimum distance between the points in the tracked and ground truth end-systolic meshes. The DSC describes the overlap in LA body segmentation between the tracked and ground truth RV segmentations in end-systole. Equal weighting was used to combine the ASD, DHD and DSC errors for identifying the optimal hyperparameter (SW, BE) combination. The change in LA shape from end-diastole to end-systole was evaluated as this involves the maximal chamber expansion during the cardiac cycle and is therefore likely to produce the largest errors in LA tracking accuracy. Using the optimised TSFFD configuration, the authors measured LA-LS in HF patients with and without atrial fibrillation (AF) and found that LA-LS was significantly reduced in those with AF.(6) We applied the same optimised TSFFD configuration to evaluate LA motion and LA-LS in our cohort of patients with severe symptomatic AS patients using TAVI CT. The accuracy of motion tracking was checked by visual inspection of tracked contours over the cardiac cycle.

**5.3 LA-LS measurement**

Peak systolic 3D LA-LS was quantified using the mean strain of all elements over the LA endocardial surface along the longitudinal axis, relative to end-diastole. The LA endocardial mesh illustrating the direction of longitudinal strain assessment is shown in Figure 5.

**Figure 5.** Longitudinal direction on the LA endocardial surface.

Longitudinal direction for strain assessment on the LA endocardial surface.

**Abbreviations**. LA: left atrium.

**6. Workflow for measuring 3D RV global and free-wall longitudinal strain**

The TSFFD method was optimised for RV motion tracking and strain assessment on CT, based on established LV and LA methodology.(5,6) This section describes the steps for creating patient-specific RV endocardial models and the framework for TSFFD hyperparameter optimisation to calculate RV global longitudinal strain (RV-GLS) and RV free-wall longitudinal strain (RV-FWLS) from motion tracking.

**6.1 Patient-specific RV model creation: segmentation and mesh**

Measurement of RV-GLS and RV-FWLS required the creation of endocardial meshes from end-diastolic segmentations of the RV. Patient-specific 3D models of the RV endocardium were created from a CNN-based 3D whole-heart multi-label segmentation of end-diastolic CT images (t=0%) (Figure 6A).(7) The RV segmentations were extracted from the multi-label segmentation using the RV blood pool label (Figure 6B). In cases where the CNN failed to generate an output, RV segmentations were created using a semi-automatic region-growing tool and 3D interpolation in CemrgApp.(8) All RV segmentations were visually assessed for accuracy and corrected in CemrgApp if necessary. Global RV endocardial surface meshes were generated from segmentations using MIRTK (<http://mirtk.github.io/>) (Figure 6C). RV free-wall endocardial surfaces meshes were created by clipping global surface meshes at the RV insertion points along the interventricular septum to isolate the free-wall (Figure 6D). For RV motion tracking optimisation, segmentations and surface meshes of the RV were created at both end-diastole (t=0%) and end-systole (t=40%) to calculate feature tracking errors. The RV longitudinal axes were defined using manually selected landmarks on end-diastolic CT images, connecting RV apex to the tricuspid valve annulus centre of mass (TV CoM) (Figure 7). This approach was adopted based on the previously described methodology for defining RV longitudinal axis on cardiac magnetic resonance imaging.(11)

**Figure 6.** Extraction of RV endocardial segmentation and surface mesh from end-diastolic CT.


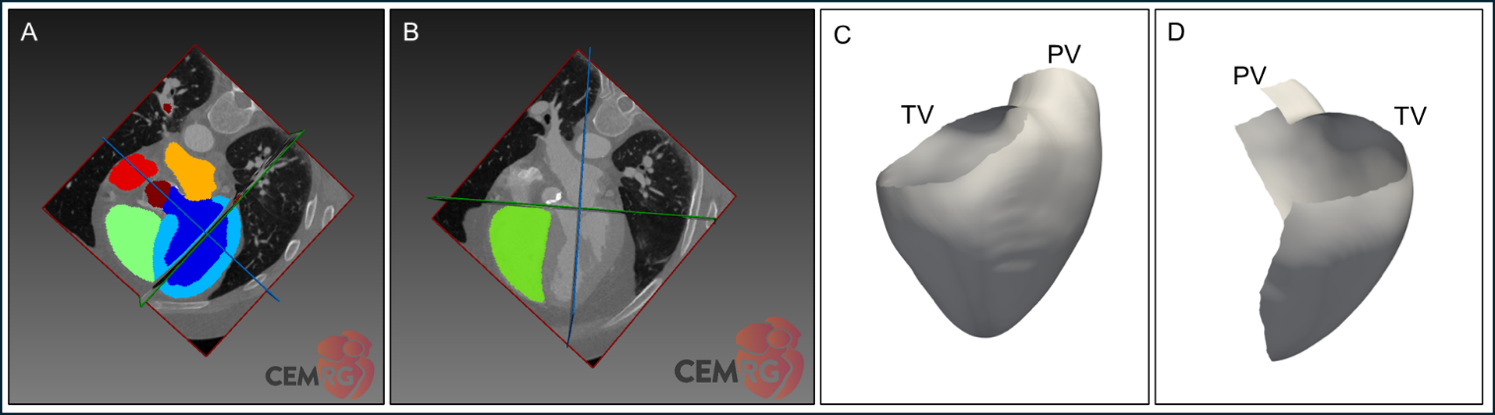


A) Multi-label segmentation output from a CNN; B) Extraction of RV segmentation using the RV blood pool label; C) RV global 3D surface mesh extraction from RV segmentation D) RV free-wall 3D surface mesh extraction.

**Abbreviations**. CT: cardiac computed tomography angiography; CNN: convolutional neural network; PV: pulmonary valve RV: right ventricle; TV: tricuspid valve 3D: 3-dimentional.

**Figure 7.** Manual landmark selection for defining RV longitudinal axis.


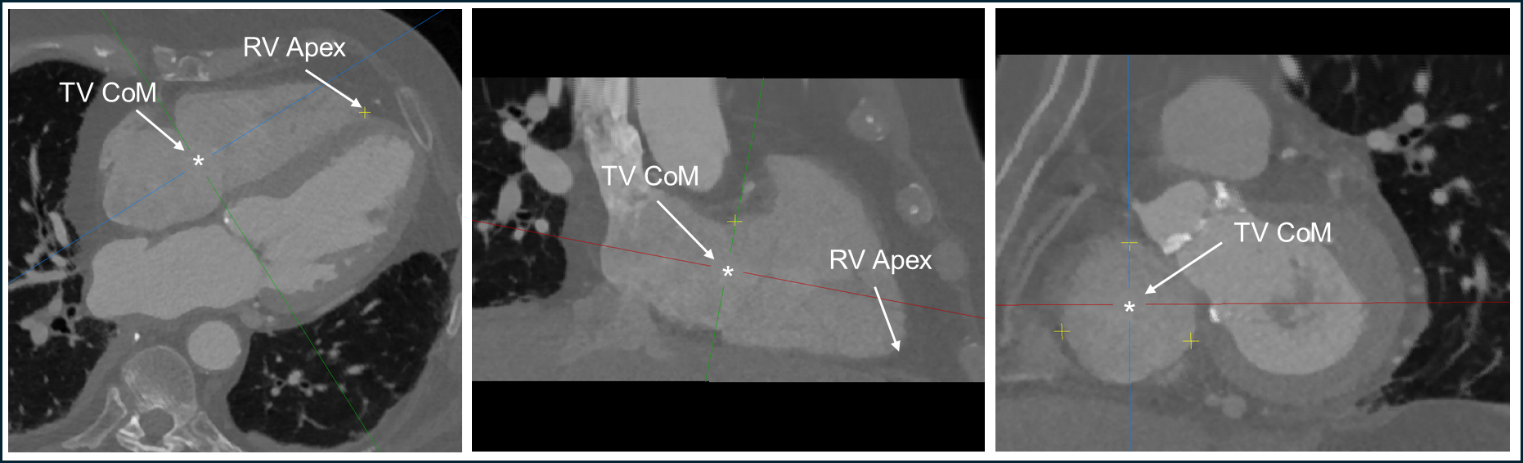


RV landmarks (green crosses) were selected in end-diastolic CT images in the RV 4-chamber, 2-chamber and short-axis views to define the RV longitudinal axes between the TV CoM and the RV apex.

**Abbreviations**. CoM: centre of mass; CT: cardiac computed tomography angiography; TV: tricuspid valve; RV: right ventricle.

**6.2 RV motion tracking optimisation**

In order to establish the optimal TSFFD hyperparameter configuration for accurate RV motion tracking, we used the methodological framework previously developed for the LA.(6) TSFFD optimisation utilised imaging data from 30 consecutive patients with severe symptomatic AS who underwent retrospectively ECG-gated CT for TAVI planning. To achieve accurate RV motion tracking, the TSFFD method was optimised with respect to two important hyperparameters (SW and BE), which are related to the sparsity constraint and the stiffness of deformation, respectively. A grid search through 25 different parameter combinations was used to identify the optimal SW and BE configuration for RV tracking. The applied hyperparameter values were based on those previously used for optimal LV and LA motion tracking on CT.(5,6) The different combinations of the SW and BE hyperparameters were systematically applied to map the deformation of RV anatomical models from end-diastole (t=0%) to end-systole (t=40%). The tracked RV meshes and segmentations were then compared with the corresponding RV meshes and segmentations created directly from the target end-systolic CT frame (t=40%), which were treated as the ground truth. Accurate motion tracking of the RV would lead to similar tracked and ground truth meshes and segmentations in end-systole. This approach follows the validation steps outlined by Sillett *et al*.(6) and was chosen over the landmark-based optimisation in Razeghi *et al*.(5) to reduce operator variability associated with manual landmark selection. The registration from end-diastole to end-systole was chosen to assess motion accuracy since this involves the largest change in RV shape and therefore should generate the largest possible tracking errors.

Using the previously described approach by Sillett *et al*.(6), the accuracy of 3D RV motion tracking was optimised using three error metrics: ASD, DHD and DSC (Figure 8). The ASD and DHD errors assess differences between the tracked and ground truth surface meshes in end-systole, while DSC measures differences between the segmentations. Lower values for ASD and DHD reflect greater similarity between the tracked and ground truth 3D RV surface meshes, indicating improved accuracy in RV motion tracking. Higher values for DSC indicate greater overlap between the tracked and ground truth segmentations, reflecting more accurate RV motion tracking. A combination of the ASD, DHD and DSC errors with equal weighting identified the optimal TSFFD hyperparameter combination. To combine the error metrics, errors were first normalised by calculating the Z-score using the following formula,

$$Z = \frac{x-\mu}{\sigma}$$

where Z is the Z-score, x is the error measurement, μ is the mean error and σ is the error standard deviation. For each hyperparameter combination, the Z-scores of each error metric were combined to give a unified error score, $S_{unified}$, as follows:

$$S_{unified}=\frac{Z_{ASD}+Z_{DHD}+1-Z_{DSC}}{3}$$

Lower values of $S_{unified}$ represent lower tracking errors relative to other hyperparameter combinations and more accurate RV feature tracking. The SW and BE values that were iterated through the TSFFD as well as the associated $S_{unified}$ scores are displayed in Figure 9. RV-optimised hyperparameters were selected by finding the minimum $S_{unified}$ score across the 30 cases. Based on TSFFD motion tracking optimisation, the hyperparameter combination with the lowest $S_{unified}$ score and therefore most accurate RV tracking was SW = 9e-3 and BE = 4e-5 (Figure 9). This hyperparameter combination yielded an $S_{unified}$ score of -0.26, which was comparable to LA motion tracking optimisation utilising this method.(6)

The performance of the optimised TSFFD motion tracking configuration for the RV was evaluated on unseen data using a hold-out test set of 10 TAVI CT scans (Figure 10). The mean ± standard deviation of the test set error values (ASD: 0.97 ± 0.12mm, DHD: 4.52 ± 2.86mm, DSC: 94.8 ± 2.8%) were comparable to those achieved with LA motion tracking optimisation and were therefore considered acceptable.(6) We applied the same optimised TSFFD configuration to evaluate RV motion and strain in the entire cohort of severe symptomatic AS patients. Motion-tracking accuracy was verified by visually inspecting the tracked contours across the cardiac cycle.

**Figure 8.** The workflow to optimise 3D RV motion tracking using CT.


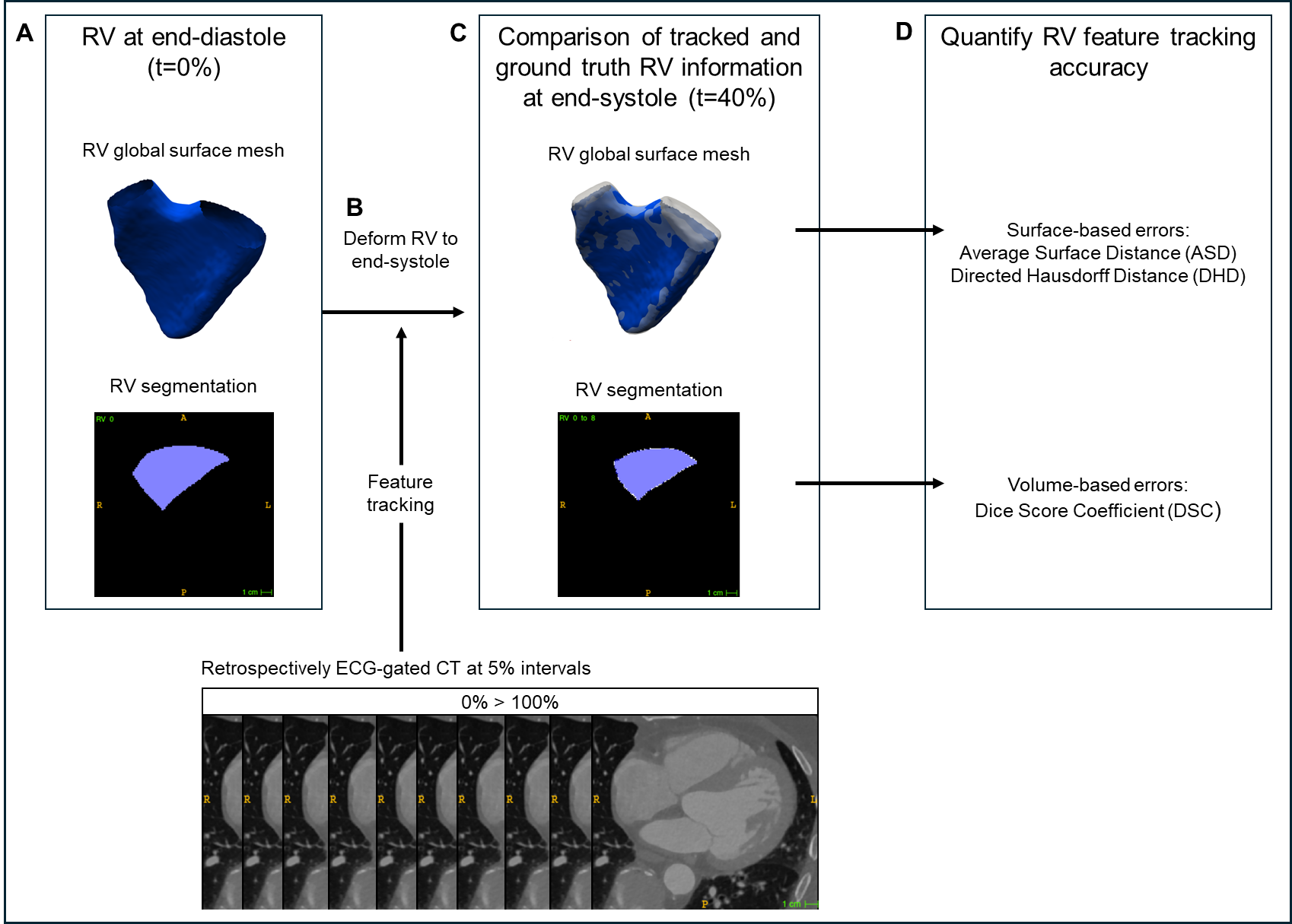


A) Anatomical information of the RV in end-diastole was captured using an endocardial surface mesh and segmentation; B) RV surface mesh and segmentation were deformed from end-diastole (t=0%) to end-systole (t=40%) using the deformation fields calculated from the TSFFD-based motion tracking on CT; C) Comparison between tracked (blue) and ground truth (transparent grey) surface meshes, as well as tracked (blue) and ground truth (white) segmentations at end-systole; D) Differences between surface meshes and segmentation at end-systole were quantified using surface-based errors (ASD and DHD) and volume-based errors (DSC).

**Abbreviations**. ASD: average surface distance; CT: cardiac computed tomography angiography; DHD: directed Hausdorff distance; DSC: dice score coefficient; RV: right ventricle.

**Figure 9.** Identification of optimal TSFFD hyperparameter combination for accurate 3D RV motion tracking.


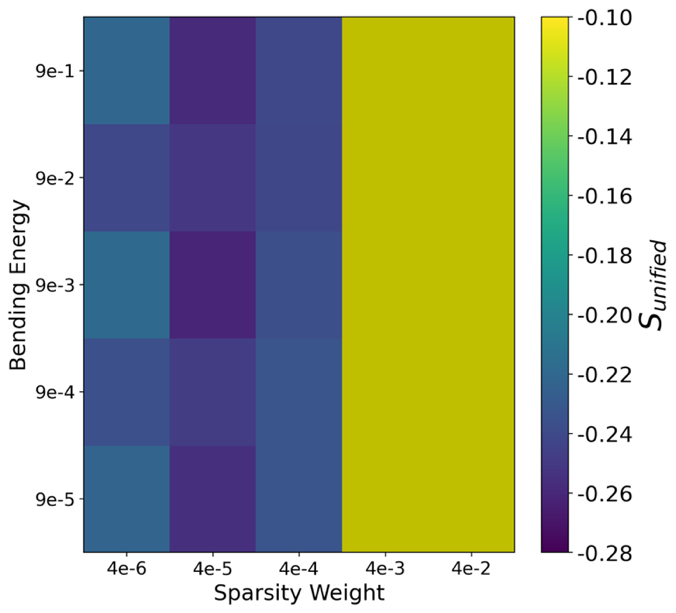


Different combinations of the sparsity weight (SW) and bending energy (BE) TSFFD hyperparameters were iterated through via a grid search. Lower values of $S_{unified}$ score indicated by darker blue shaded squares, represent more accurate RV feature tracking. The best performing SW and BE combination was 4e-5 and 9e-3, respectively.

**Figure 10.** Optimised TSFFD configuration performance on a hold-out test set of 10 TAVI CT scans.


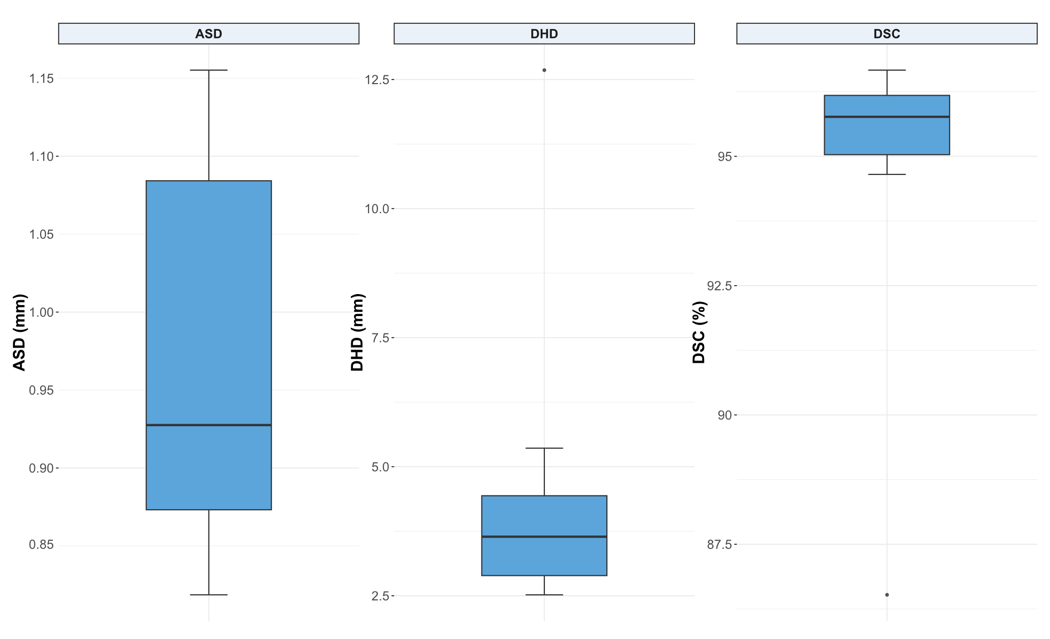


**Abbreviations**. ASD: average surface distance; DHD: directed Hausdorff distance; DSC: dice score coefficient.

**6.3 RV-GLS and RV-FWLS measurement**

Peak systolic RV-GLS and RV-FWLS were calculated as the mean strain of all elements on the global and free-wall RV endocardial surface along the longitudinal axis, relative to end-diastole. Figure 11 shows a global RV endocardial mesh with illustrated longitudinal direction for strain assessment.

**Figure 11.** Longitudinal direction on the global RV endocardial surface.

Longitudinal direction for strain assessment on the RV global endocardial surface.

**Abbreviations.** RV: right ventricle.

**References**

1. Voigt JU, Mălăescu GG, Haugaa K, Badano L. How to do LA strain. Eur Heart J Cardiovasc Imaging. 2020 Jul 1;21(7):715–7.

2. Tobon-Gomez C, De Craene M, McLeod K, Tautz L, Shi W, Hennemuth A, et al. Benchmarking framework for myocardial tracking and deformation algorithms: an open access database. Med Image Anal. 2013 Aug;17(6):632–48.

3. Rueckert D, Sonoda LI, Hayes C, Hill DL, Leach MO, Hawkes DJ. Nonrigid registration using free-form deformations: application to breast MR images. IEEE Trans Med Imaging. 1999 Aug;18(8):712–21.

4. Shi W, Jantsch M, Aljabar P, Pizarro L, Bai W, Wang H, et al. Temporal sparse free-form deformations. Med Image Anal. 2013 Oct;17(7):779–89.

5. Razeghi O, Heinrich M, Fastl TE, Corrado C, Karim R, De Vecchi A, et al. Hyperparameter optimisation and validation of registration algorithms for measuring regional ventricular deformation using retrospective gated computed tomography images. Sci Rep. 2021 Mar 11;11(1):5718.

6. Sillett C, Razeghi O, Lee AWC, Solis Lemus JA, Roney C, Mannina C, et al. A three-dimensional left atrial motion estimation from retrospective gated computed tomography: application in heart failure patients with atrial fibrillation. Front Cardiovasc Med. 2024;11:1359715.

7. Xu H, Niederer SA, Williams SE, Newby DE, Williams MC, Young AA. Whole Heart Anatomical Refinement from CCTA Using Extrapolation and Parcellation. In: Ennis DB, Perotti LE, Wang VY, editors. Functional Imaging and Modeling of the Heart. Cham: Springer International Publishing; 2021. p. 63–70.

8. Razeghi O, Solís-Lemus JA, Lee AWC, Karim R, Corrado C, Roney CH, et al. CemrgApp: An interactive medical imaging application with image processing, computer vision, and machine learning toolkits for cardiovascular research. SoftwareX. 2020;12:100570.

9. Solís-Lemus JA, Baptiste T, Barrows R, Sillett C, Gharaviri A, Raffaele G, et al. Evaluation of an open-source pipeline to create patient-specific left atrial models: A reproducibility study. Comput Biol Med. 2023 Aug;162:107009.

10. Sillett C, Razeghi O, Strocchi M, Roney CH, O’Brien H, Ennis DB, et al. Optimisation of Left Atrial Feature Tracking Using Retrospective Gated Computed Tomography Images. Funct Imaging Model Heart. 2021 Jun;12738:71–83.

11. Arenja N, Riffel JH, Djiokou CN, Andre F, Fritz T, Halder M, et al. Right ventricular long axis strain-validation of a novel parameter in non-ischemic dilated cardiomyopathy using standard cardiac magnetic resonance imaging. Eur J Radiol. 2016 Jul;85(7):1322–8.
